# *Bacillus subtilis* DinG 3′⟶5′ Exo(ribo)nuclease: A Helpmate to Mitigate Replication Stress

**DOI:** 10.3390/ijms26199681

**Published:** 2025-10-04

**Authors:** Begoña Carrasco, Rubén Torres, María López-Sanz, Rogelio Hernández-Tamayo, Peter L. Graumann, Juan C. Alonso

**Affiliations:** 1 Department of Microbial Biotechnology, Centro Nacional de Biotecnología (CNB-CSIC), Calle Darwin 3, 28049 Madrid, Spain; bcarrasc@cnb.csic.es (B.C.); rtorres@cnb.csic.es (R.T.); mlopez@cnb.csic.es (M.L.-S.); 2 Center for Synthetic Microbiology (SYNMIKRO), Karl-von-Frisch-Straße 14, 35043 Marburg, Germany; rogelio.hernandez@synmikro.uni-marburg.de (R.H.-T.); graumanp@uni-marburg.de (P.L.G.); 3 Max Planck Institute for Terrestrial Microbiology, Karl-von-Frisch-Straße 10, 35043 Marburg, Germany; 4 Department of Chemistry, University of Marburg, Hans-Meerwein-Straße 4, 35032 Marburg, Germany

**Keywords:** replication fork stalling, R-loops, RecA, RNA polymerase, RnhC, PolA

## Abstract

*Bacillus subtilis* DinG/XPD-like paralogues, DinG and YpvA, have been implicated in overcoming replication stress. DinG possesses a DEDD exonuclease and DNA helicase domains, whereas YpvA lacks the DEDD exonuclease domain. We report that DinG·Mg^2+^ (hereafter referred to as DinG) degrades linear single-stranded (lss) DNA with 3′→5′ polarity and binds lssDNA with higher affinity than its exonuclease-deficient mutant DinG D10A E12A. DinG’s ssDNA-dependent ATPase activity neither stimulates nor inhibits DNA degradation. When bound to the 3′-end of forked DNA, DinG destabilises and degrades the substrate; however, in the presence of ATP, DinG dissociates before reaching the duplex junction. DinG degrades the RNA strand within RNA–DNA hybrids but does not cleave lssRNA unless complexed with Mn^2+^. DinG removes genomic R-loops, as RnhC and PcrA do. DinG physically interacts with RecA and PolA and functions in the same pathway as translesion synthesis (TLS) DNA polymerases (DNAPs) to respond to both spontaneous and methyl methanesulphonate (MMS)-induced mutagenesis. DinG-mGold forms spontaneous foci at or near replication forks, which become enriched following MMS or rifampicin treatment. We propose that DinG contributes to mitigating replication stress by degrading R-loop barriers and facilitating TLS, potentially via RecA-linked mechanisms.

## 1. Introduction

*Escherichia coli*, the best-characterised bacterium of the Pseudomonadota (also known as Proteobacteria) phylum, encodes two members of the damage-inducible *Xeroderma pigmentosum* complementation group D (XPD)-DinG family of enzymes [1]—DinG and YoaA—which share identity with the DNA helicases Rad3 from *Saccharomyces cerevisiae* and XPD from humans (Supplementary Figure S1A) [2,3,4,5]. DinG*_Eco_* (712 amino acids [aa]) and YoaA*_Eco_* (636 aa) each possess two helicase domains (HD1 and HD2), an Arch domain, and an iron–sulphur (FeS) cluster-binding motif [6]. DinG*_Eco_* binds single-stranded (ss) DNA and unwinds diverse DNA structures—including overhangs, flap structures, forks, D-loops, and R-loops—in the 5′⟶3′ direction [4,6,7]. In contrast, YoaA*_Eco_*, in association with the DNA polymerase (DNAP) clamp loader subunit HolC/χ*_Eco_*, unwinds forked duplex DNA with both 3′ and 5′ overhangs [8].

*Bacillus subtilis*, a model bacterium of the phylum Bacillota (also known as Firmicutes), which is phylogenetically separated from *E. coli* by >2000 million years, encodes two discrete DinG/XPD-like paralogues—DinG and YpvA—that are not damage-inducible [9]. Unless stated otherwise, all genes and gene products referenced are of *B. subtilis* origin. DinG (931 aa long) has an N-terminal DEDD exonuclease-like domain, two helicase domains (HD1 and HD2), and an Arch domain, but lacks the cysteine residues required to coordinate the crucial iron–sulphur (FeS) cluster-binding motif (Supplementary Figure S1A–C) [1]. *Staphylococcus aureus* DinG (DinG*_Sau_*, 897 aa long), which shares ~29% sequence identity with DinG, is a *bona fide* 3′⟶5′ exonuclease active on ssDNA and RNA substrates [10]. YpvA (641 aa long) has the same overall domain architecture as DinG*_Eco_* and YoaA*_Eco_*, sharing ~25% sequence identity with them. The biochemical activities of DinG and YpvA, and their respective contributions to overcoming replication stress, remain unknown. The predicted structures of DinG (P54394) and YpvA (P50831), modelled using AlphaFold2 and RoseTTAfold, exhibit highly conserved folds with those of DinG*_Sau_*-ssDNA-Ca^2+^ (8ZEF, 9II8), and of DinG*_Eco_*-ssDNA and DinG_Eco_-ssDNA-ADPBeF3 (6FWR, 6FWS), respectively [6,11] (https://www.uniprot.org/uniprotkb/).

During bacterial DNA synthesis, replicative DNA polymerase (DNAP) frequently encounters endogenous threats—such as collisions with an array of RNA polymerases (RNAPs) elongation complexes and/or R-loops that lead to replication–transcription conflicts (RTCs), mis-incorporated rNMPs, and DNA lesions (e.g., oxidised bases)—that trigger local replication stress reviewed in [12,13,14]. Unlike *E. coli*, the *B. subtilis* replicative DNAP transiently pauses upon colliding with an array of RNAPs transcribing highly expressed genes—such as *rrn* loci—in co-directional (CD) orientation [15], and in a significant fraction of cells (>40%), the replisome spontaneously disassembles [16]. Live-cell fluorescent microscopy of unstressed cells has revealed that the recombinase RecA, the helicase PcrA (a functional homologue of UvrD*_Eco_*), and the endo-exoribonuclease RnhC (also known as RNase HIII, a functional counterpart of RnhA*_Eco_*) form spontaneous foci. These foci colocalise with DnaX and are enriched at *rrn* loci [17,18,19,20,21]. Furthermore, ~30% of translesion synthesis (TLS) DNAP molecules—including the family-A PolA (also known as Pol I) and family-Y PolY1 (also known as YqjH)—also form spontaneous foci in unstressed cells. Their recruitment indicates ongoing bypass of endogenous oxidative base lesions and suggests that TLS polymerases act under basal replication stress conditions [21,22]. Upon exposure to elevated exogenous threats, both local and general replication stress responses are triggered, including SOS induction, which elevates the expression of RecA, the family-Y TLS DNAP PolY2 (also known as YqjW), and, to a lesser extent, the essential C-family TLS DNAP DnaE [9,23].

In this study, we combined genetic, cytological, and biochemical approaches to elucidate the role of DinG in the response to DNA replication stress. In a prophage-free background, a Δ*dinG* mutation produced no detectable phenotype following exposure to the DNA damaging agents MMS, H_2_O_2_, or mitomycin C (MMC). In contrast, the inactivation of YpvA rendered cells more proficient in DNA repair than either wild type (wt) or Δ*dinG* cells. Loss of DinG reduced both untargeted and MMS-induced mutagenesis, and DinG functions in the same pathway as the TLS DNAPs, suggesting that it assists bipartite TLS DNAPs (PolA and PolY1 or PolY2). A DinG-mGold fusion formed spontaneous foci enriched at or near DnaX, and foci abundance increased upon treatment with MMS or rifampicin (Rif).

We constructed DinG variants carrying mutations in either the ExoI subdomain (DinG D10A E12A) or the ATP-binding motif I (also known as Walker A box, DinG K290A) (Supplementary Figure S1A,B). wt DinG, DinG D10A E12A, and DinG K290A proteins were purified using a similar protocol and characterised biochemically. Circular (cssDNA) or lssDNA stimulated the ATPase activity of both DinG and DinG D10A E12A, but not that of DinG K290A. DinG and DinG K290A degraded lssDNA with 3′→5′ polarity, whereas DinG D10A E12A did not. Specifically, at limiting protein-to-DNA ratios, DinG degraded lssDNA yielding 11 ± 2-nucleotides (nt) products, irrespective of ATP. At near-stoichiometric ratios, the products were shortened to 6 ± 1 nt. This degradation occurred in a sequence-independent manner. DinG degraded the 3′-tail of forked DNA but had limited activity on duplex DNA, while DinG·ATP dissociated before reaching the duplex junction. DinG degraded the nascent strand of DNA structures mimicking stalled forks and the complementary strand of both 3′-overhang and 5′-overgang DNA, albeit with lower efficiency. It also degraded, with low efficiency, the RNA strand of RNA–DNA hybrids, but did not cleave lssRNA, indicating a preference for RNA–DNA hybrids (R-loops) over lssRNA. Consistent with this, DinG removed genomic R-loops, as RnhC and PcrA do. Together, these results demonstrate that the 3′→5′ exonuclease activity of DinG helps to overcome replication fork barriers such as R-loops and promotes TLS bypass by a poorly understood mechanism, to resume DNA synthesis. Functional redundancies likely mask the analysis of the contribution of DinG to replication in vivo.

## 2. Results and Discussion

### 2.1. DinG Cannot Cleave Circular ssDNA or dsDNA, But Degrades Nicked dsDNA

To investigate the role(s) of DinG, we overexpressed and purified the wt enzyme as well as the mutant variants, DinG D10A E12A and DinG K290A, following the same protocol (see Section 4). The native 106 kDa DinG, DinG D10A E12A, and DinG K290A proteins were ~99% pure, as assessed by SDS-polyacrylamide gel electrophoresis (PAGE) (Supplementary Figure S2A) and quantitative analysis of peptide mass fingerprinting.

In a limited number of Bacillota species, DinG-like proteins have been reported to possess an N-terminal putative endonuclease domain [24]. To determine whether DinG in its Mg^2+^-bound form exhibits exonuclease and/or endonuclease activity on plasmid-sized DNA, we used a 3199 nt cssDNA and a 3199 base pair (bp) supercoiled (scDNA) pGEM3 Zf(+) DNA (50% dC + dG content) and saturating protein concentrations (a DinG monomer/57 to 28 nt or bp). No endonuclease activity was detected for DinG, DinG D10A E12A, or DinG K290A (Supplementary Figure S2B, lanes 2–7 and 9–14).

The minor subfraction of open circular dsDNA (ocDNA) present in the scDNA preparation was degraded by DinG or DinG K290A, but not by the exonuclease-deficient DinG D10A E12A variant (Supplementary Figure S2B, lanes 11–12 vs. 9–10 and 13–14). These results indicate that: (i) DinG lacks detectable endonuclease activity on cssDNA or scDNA under the tested conditions; and (ii) a large excess of DinG or DinG K290A can degrade the nicked substrate in a sequence independent manner, but mutation of the ExoI subdomain (DinG D10A E12A) abolishes degradation. Although we cannot exclude a model in which DinG initiates cleavage on the strand opposite a nick and subsequently degrades ldsDNA, we consider this scenario unlikely (see below).

### 2.2. Saturating Concentrations of DinG Are Required to Degrade Linear dsDNA

To test whether DinG degrades linear double-stranded (lds) DNA bearing 3′ and 5′ 4 nt overhangs, the 3199 bp pGEM3 Zf(+) scDNA was linearised with KpnI or EcoRI, respectively. Under saturating DinG conditions (one DinG monomer/115 to 28 bp), the enzyme partially degraded (400 to 800 nt) both ldsDNA substrates from both ends after a 15 min incubation at 37 °C (Supplementary Figure S2C, lanes 3–5 and 7–9).

DinG is a low-abundance protein in vivo (∼45 monomers/cell, ∼60 nM) [25]. We examined its activity using stoichiometric concentrations of DinG relative to the DNA substrate to provide insight into its physiological role. At sub- to stoichiometric concentrations of DinG (0.87, 1.75, and 3.5 nM, ~0.25 to ~1 DinG monomer/ldsDNA molecule), no appreciable degradation was observed after 15 min of incubation at 37 °C (Supplementary Figure S2C, lanes 12–14 and 16–18). These findings indicate that DinG supports only limited degradation of duplex DNA with 4 nt overhangs when present in excess, but is inactive at stoichiometric concentrations, which may reflect its physiological behaviour.

### 2.3. DinG Preferentially Degrades lssDNA over lssRNA, with 3′⟶5′ Polarity

DinG*_Sau_* preferentially degrades lssRNA over lssDNA at 37 °C, although this may reflect a sequence preference, as the tested substrates differed in dC + dG content [10]. Notably, DinG*_Sau_* shows a preference for polypyrimidine substrates, and stoichiometric concentrations completely cleaved a 20 nt linear poly(dT_20_) lssDNA to 1 nt products within 30 min at 37 °C [11]. To compare substrate specificity with DinG, which shares only ~29% sequence identity with DinG*_Sau_*, we synthesised 38 nt linear ssDNA and ssRNA oligonucleotides of identical sequence (except for U/T) and 5′-end-labelled them to generate γ^32^P-lssDNA_38_ and γ^32^P-lssRNA_38_ (Supplementary Figure S3; Table S2).

**Figure 1:**
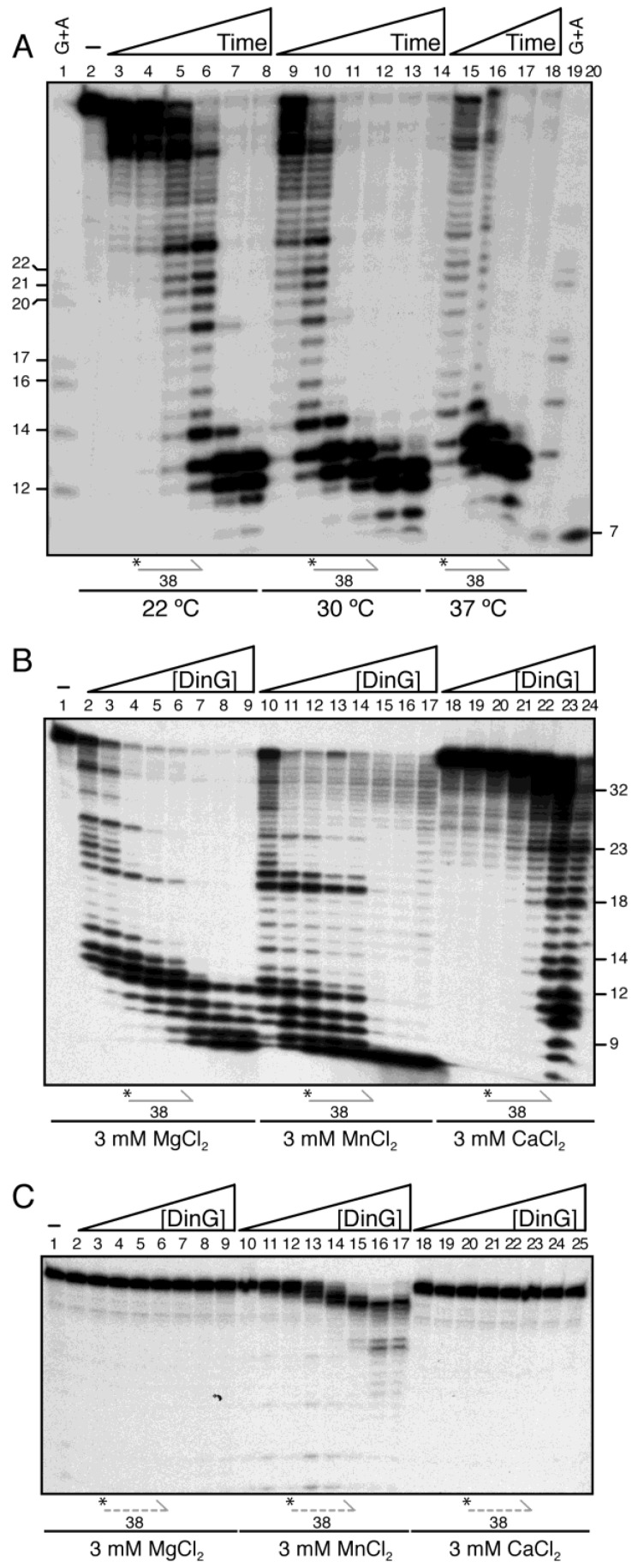
DinG preferentially degrades lssDNA over lssRNA. (**A**) The γ^32^P-5′-^32^P-lssDNA_38_ (5 nM in DNA molecules) was incubated with 0.5 nM DinG for a variable time (0.5, 1, 2.5, 5, 15, and 30 min [lanes 3–8 and 9–14] or 0.5, 1, 2.5, and 5 min [lanes 15–18]) at 22 °C (lanes 3–8), 30 °C (lanes 9–14), or 37 °C (lanes 15–18). “G + A”, mobility markers (lanes 1 and 19). In lane 20, a radiolabelled oligonucleotide co-migrating with the 7 nt marker is shown. (**B**) The γ^32^P-5′-lssDNA_38_ or (**C**) γ^32^P-5′-lssRNA_38_ (5 nM in DNA molecules) was incubated with increasing DinG concentrations (0.3–50 nM) for 15 min at 22 °C in buffer D (lanes 1–9), or in buffer D in which Mg^2+^ was replaced by Mn^2+^ (lanes 10–17), or Ca^2+^ (lanes 18–25). Products were separated under denaturing conditions. “–”, no protein added; grey line, 5′-end labelled strand; solid line, DNA; dashed line, RNA; “*”, labelled nucleotide; arrowhead, 3′-end; and number, length of each strand (in nt). Relevant molecule lengths are indicated (in nt). All reactions were repeated at least three times with consistent results, and representative gel images are shown.

Limiting DinG (0.5 nM), relative to lssDNA (5 nM in DNA molecules), rapidly degraded >65% of the γ^32^P-lssDNA_38_ substrate in <30 s at 37 °C, generating discrete intermediates detectable under denaturing conditions (Figure 1A, lane 15). After 1 min, 12 ± 2 nt products accumulated, and with prolonged incubation, an additional nucleotide was trimmed to yield 11 ± 1 nt products (Figure 1A, lane 16 vs. 18). Since the oligonucleotide is 5′-labelled and DinG lacks endonuclease activity, these intermediates represent progressive 3′⟶5 exonucleolytic degradation of lssDNA from the 3′-end.

To examine the stepwise degradation of lssDNA, DinG was incubated with the substrate at reduced temperatures (22 °C and 30 °C). Within a 30 s incubation at 22 °C, ~50% of the substrate was degraded, accumulating 37 to 34 nt long intermediates (Figure 1A, lane 3). With 2.5 min of incubation, intermediates progressively shortened, ultimately yielding 13 ± 1 nt products via a low-processivity mechanism (Figure 1A, lane 5). After 5 min, the major products were 12 ± 1 nt, and by 30 min, an additional nucleotide was removed, yielding 11 ± 1 nt products (Figure 1A, lane 6 vs. 7).

The observed slow trimming could reflect suboptimal catalytic turnover or inefficient Mg^2+^ coordination. To test this, the γ^32^P-lssDNA_38_ was incubated for 15 min at 22 °C with increasing DinG concentrations (0.3 to 50 nM) in buffer D, or in buffer D in which the Mg^2+^ metal ion (3 mM MgCl_2_) was substituted with Mn^2+^ or Ca^2+^ ions. With Mg^2+^, limiting DinG produced discrete intermediates and 11 ± 2 nt end-products (Figure 1B, lanes 2–3). Just above stoichiometry, products shortened further to 11-6 nt (Figure 1B, lanes 7–9), but no shorter fragments were detected even with a 10-fold DinG excess. With Mn^2+^, intermediate lengths decreased continuously, and 6 ± 1 nt products accumulated just above stoichiometric DinG concentrations (Figure 1B, lanes 10–14 vs. 15–17). By contrast, with Ca^2+^, an excess of the enzyme (~5 DinG·Ca^2+^/ssDNA molecule) was required to achieve cleavage (Supplementary Figure S1B, lane 23).

A different pattern emerged when γ^32^P-lssDNA_38_ was replaced by γ^32^P-lssRNA_38_. In the presence of Mg^2+^ or Ca^2+^, no degradation was detected (Figure 1C, lanes 2–9 and 18–25). With Mn^2+^ modest trimming occurred only just above stoichiometric levels (1.2 DinG monomers/lssRNA molecule), producing 26 to 24 nt intermediates at higher DinG amounts (5 to 10 DinG monomers/lssRNA molecule) (Figure 1C, lane 14 vs. 16–17).

These results suggest three mechanistic insights into DinG’s nuclease activity: (i) two-phase reaction at limiting enzyme concentrations—DinG may trim a few nucleotides within 60 s at 22 °C, then likely undergoes a conformational switch that reduces dissociation and enters a low-processivity 3′⟶5 mode, producing 13 to 10 nt products (Figure 1A, lanes 3–4 vs. 7–8, and Figure 1B, lanes 2–7); (ii) at stoichiometric DinG–lssDNA ratios, protein–protein interactions may stabilise substrate binding, allowing further trimming to 8 to 6 nt products; and 6 ± 1 nt products in Mn^2+^ (Figure 1B, lanes 7–9 vs. 15–17); and (iii) only saturating DinG·Mn^2+^ degraded the γ^32^P-lssRNA_38_ substrate, albeit with low efficiency when compared with lssDNA degradation (Figure 1B vs. Figure 1C). The apparent differences between DinG and DinG*_Sau_* could reflect genuine differences in substrate preference or arise from residual sequences (traces of the tobacco etch virus cleavage site) present in the DinG*_Sau_* protein after His-tag removal [10,11].

Because prior studies on DinG*_Eco_* and DinG*_Sau_* used MgCl_2_ [4,10], and Mg^2+^ is the preferred cofactor for RnhC and PcrA activity on RNA–DNA hybrids [26,27], Mg^2+^ was used in all subsequent assays.

### 2.4. ssDNA Stimulates the ATPase Activity of DinG and DinG D10A E12A

DinG contains conserved ATP-binding and hydrolysis domains (Supplementary Figure S1C) [1]. To quantify its ATPase activity, we monitored the steady-state rate of ATP hydrolysis at 37 °C for 30 min in buffer D containing 5 mM ATP and an ATP regeneration system, with DNA added where indicated.

**Figure 2:**
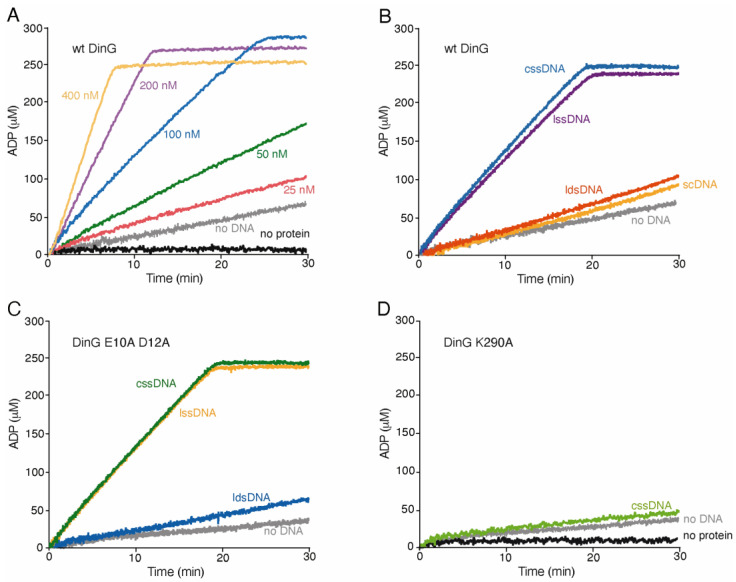
DinG exhibits ssDNA-dependent ATPase activity. (**A**) Increasing concentrations of DinG (25–400 nM) were incubated with a fixed concentration of cssDNA (10 μM in nt molecules) in buffer D containing 5 mM ATP and an ATP regeneration system. ATPase activity was measured over 30 min at 37 °C. Fixed wt DinG (**B**), DinG D10A E12A (**C**) or DinG K290A (**D**) (100 nM) was incubated with the indicated substrate under the same conditions. “no protein” refers to a mock reaction, and “no DNA” indicates that DNA was not added. All reactions were repeated at least three times with similar results, and representative graphs are shown.

In the absence of DNA, DinG exhibited a basal ATPase turnover rate above background levels, with a K_cat_ of 22 ± 2.2 min^−1^ (mean ± SEM) (Figure 2A,B). The DinG K290A and DinG D10A E12A mutants showed slightly reduced basal rates, with K_cat_ values of 11 ± 0.9 min^−1^ and 8 ± 0.6 min^−1^, respectively (Figure 2C,D). We cannot completely exclude the possibility that trace ssDNA or ATPase contaminants in the protein preparations contribute to the observed basal rates. Nevertheless, the maximal basal ATP hydrolysis rate for DinG was comparable to that of DinG*_Sau_* and lower than that reported for DinG*_Eco_* see [4,10].

When increasing concentrations of DinG (25 to 400 nM) or fixed amounts of DinG or DinG D10A E12A (100 nM) were incubated with 3199 nt cssDNA (10 μM in nt), the steady-state rate of ATP hydrolysis was significantly stimulated, 6- and 15-fold, reaching K_cat_ values of 125 ± 5.3 min^−1^ and 122 ± 9.1 min^−1^, respectively, with no detectable lag phase (Figure 2A–C). In contrast, the ATPase activity of DinG K290A was not stimulated by cssDNA (Figure 2D). These results indicate that cssDNA is a potent activator of DinG’s ATPase activity, and that the ExoI subdomain is dispensable for this stimulation, while the ATP-binding motif I is critical.

### 2.5. The ATPase Activity of DinG Is Not Stimulated by Either lssDNA Degradation or Duplex DNA

To understand whether the ATPase and exonuclease activities of DinG affect each other, DinG or DinG D10A E12A (100 nM) was incubated with either 3199 nt long cssDNA or lssDNA, and ATP hydrolysis was measured. The maximal rate of ATP hydrolysis by DinG was similar in the presence of either 3′-tailed lssDNA or cssDNA, regardless of the fact that the former is degradable by DinG whereas the latter is not, as it lacks a free 3′-end (Figure 2B). Additionally, the DinG D10A E12A mutant, which is impaired in exonuclease activity (see below), hydrolyses ATP with similar efficiency with both DNA substrates (Figure 2C). It is likely that the exonuclease activity of DinG does not affect the pattern of DinG-mediated ATP degradation.

Next, we tested whether ATP hydrolysis facilitates DinG dissociation from secondary structures present in ssDNA or from duplex DNA. To test the hypothesis, the ATPase activity of wt DinG (100 nM) was measured in the presence of 3199 bp ldsDNA or scDNA (10 μM in bp). Both ldsDNA and scDNA marginally stimulated the ATPase rate of DinG (K_cat_ of 36 ± 5.3 min^−1^ and 31 ± 5.5 min^−1^, respectively) when compared with the absence of DNA (K_cat_ of 22 ± 2.2 min^−1^) (Figure 2B). Similar results were observed when DinG D10A E12A was incubated with ldsDNA (Figure 2C). Therefore, it is unlikely that ATP hydrolysis stimulates DinG dissociation from dsDNA.

### 2.6. DinG Engages 3′-Ends, and ATP Slightly Attenuates lssDNA Degradation

Two models could explain how DinG achieves unidirectional 3′→5′ exonuclease activity on ssDNA: (a) direct docking at the 3′-terminus followed by successive nucleotide removal; or (b) initial binding to the lssDNA with ATP-dependent (or independent) translocation toward the 3′-end before cleavage. To distinguish between these possibilities, we performed a streptavidin (SA) block assay using a 45 nt oligomer radiolabelled at its 5′-end and bearing a single biotin at the second internucleotide position from the 3′-end (3′-Biotin-γ^32^P-lssDNA_45_) (Supplementary Figure S3 and Table S2). With this assay, we also tested whether ATP regulates the DNA degradation rate.

**Figure 3:**
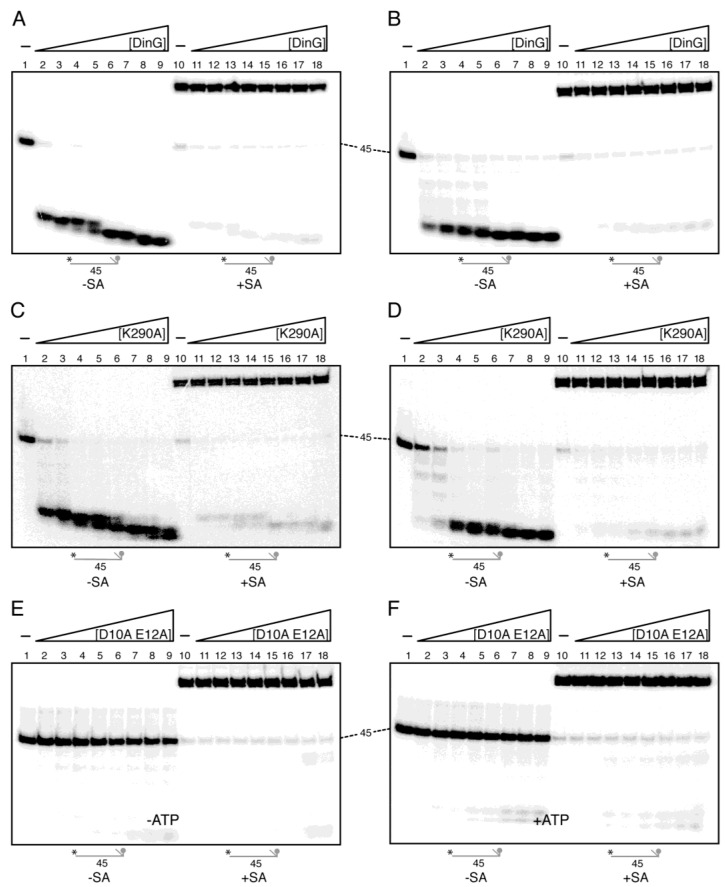
DinG binds and degrades lssDNA in the 3′⟶5′ direction but cannot displace a streptavidin–biotin block at the 3′-end. (**A**–**F**) The 3′-Biotin γ^32^P-lssDNA_45_ (0.5 nM in DNA molecules) was incubated with increasing concentrations of DinG (0.09–12 nM (**A**,**B**)), DinG K290A (0.09–12 nM (**C**) or 0.02–3.1 nM (**D**)), or DinG D10A E12A (0.7–100 nM (**E**,**F**)) for 5 min at 37 °C in buffer D, either lacking (**A**,**C**,**E**) or containing 2.5 mM ATP (**B**,**D**,**F**). Then, the reactions were deproteinised, and the products were separated by native PAGE. When indicated ((**A**–**F**), lanes 11–18), the 3′-Biotin γ^32^P-lssDNA_45_ was pre-incubated with saturating SA concentrations (2.5 nM) to block the 3′ end before enzyme addition. The preformed SA-3′-Biotin γ^32^P-lssDNA_45_ complex was then incubated with increasing concentrations of the corresponding enzyme. “–”, no protein added; grey line, 5′-end labelled strand; solid line, DNA; “*”, labelled nucleotide; arrowhead, 3′-end; and number, length of each strand (in nt); dot, dT_44_ biotin. All reactions were performed at least three times with consistent results, and representative gel images are shown.

First, the 3′-Biotin-γ^32^P-lssDNA_45_ (0.5 nM in DNA molecules) was incubated with increasing DinG concentrations for 5 min at 37 °C in buffer D in the absence of SA. One DinG monomer per ~5 lssDNA_45_ molecules was sufficient to degrade >90% of the substrate (Figure 3A, lane 2), suggesting that the biotin does not impair DinG degradation. At near-stoichiometric ratios, DinG generated two discrete fragments, and at concentrations above stoichiometry (∼3 DinG/lssDNA_45_ molecule), the intermediates were further trimmed (Figure 3A, lanes 5–9).

DinG is an ATP-independent exonuclease. To test whether ATP impacts degradation, parallel reactions containing 2.5 mM ATP were performed. Limiting DinG still degraded >70% of the substrate after a 5 min incubation at 37 °C, producing the same end-products as in the absence of ATP. This indicates only a slight ATP-dependent inhibition of exonuclease activity compared with the apo enzyme (Figure 3A vs. Figure 3B, lanes 2–9). By contrast, DinG*_Sau_* fails to degrade lssDNA in the presence of 1 mM ATP during a 60 min reaction at 37 °C [10]. The molecular basis for these divergent ATP effects on DinG orthologues remains unknown.

DinG K290A produced a degradation profile similar to that of the apo enzyme (Figure 3C, lanes 2–9), confirming that the biotin does not impede the 3′→5′ exonuclease activity of the protein. To test whether ATP indirectly affects cleavage—possibly by chelating Mg^+2^—we incubated the substrate with substoichiometric DinG K290A in the presence of 2.5 mM ATP. Even at a ratio of one enzyme per ~25 DNA molecules, DinG K290A degraded ~50% of the substrate, higher enzyme concentrations yielded the same intermediates and end-products as apo DinG (Figure 3D, lanes 4–9 vs. Figure 3B, lanes 2–9). These results suggest that: (i) ATP is neither necessary for DinG-mediated degradation of γ^32^P-lssDNA_45_ nor for fuelling translocation along lssDNA; (ii) the exonuclease and ATPase activities of DinG are not mutually exclusive (Figure 3A,C); and (iii) ATP slightly reduces, if at all, the exonuclease activity of DinG on the 3′-Biotin γ^32^P-lssDNA_45_ substrate. DinG*_Sau_* binds lssDNA with ~three-fold lower affinity in the presence of 2.5 mM ATP [11]; whether ATP reduces the binding of DinG to lssDNA remains unknown.

Next, we analysed whether DinG could displace SA from the 3′-end. Preformed SA-3′-Biotin γ^32^P-lssDNA_45_ complexes were generated by incubating the biotinylated substrate with a five-fold molar excess of SA, which retards the DNA on native PAGE (Figure 3A–F, lane 10). In both ATP-free and ATP-containing reactions, neither wt DinG nor DinG K290A degraded the SA-3′-Biotin γ^32^P-lssDNA_45_, although both enzymes efficiently trimmed any residual SA-free γ^32^P-lssDNA_45_ (Figure 3A–D, lanes 11–18). These results indicate that: (i) SA bound at the biotinylated 3′-end sterically prevents DinG from initiating DNA degradation; (ii) DinG, whether ATP-bound or apo, cannot displace an SA-biotin block; and (iii) exonucleolytic entry strictly requires a free 3′ terminus, suggesting that DinG recognises the 3′ end of the DNA substrate to ensure 3′→5′ exonuclease activity. While this manuscript was in preparation, it was reported that DinG*_Sau_* poorly degraded a poly(dT_20_) substrate carrying 6-carboxfluorescein at the 3′-end [11], reinforcing that DinG acts as a 3′→5′ exonuclease and is highly specific for unblocked ssDNA ends.

To rule out contamination by *E. coli* exonucleases, the 3′-Biotin γ^32^P-lssDNA_45_ substrate was incubated with the ExoI—deficient DinG D10A E12A variant, purified using a protocol similar to that for wt DinG. No degradation was observed even in the presence of ~1000-fold excess of DinG D10A E12A compared with wt DinG, both in the absence or presence of ATP (Figure 3E,F). This demonstrates that exonuclease activity can be solely attributed to, and is intrinsic to, the DinG polypeptide.

### 2.7. DinG Binds lssDNA with High Affinity, But Not DinG D10A E12A

Electrophoretic mobility shift assays (EMSAs) were used to quantify the binding of DinG to the 3′-Biotin γ^32^P-lssDNA_45_. Reactions containing 0.5 nM DNA (in DNA molecules) and increasing enzyme concentrations were incubated for 5 min at 37 °C in buffer D and resolved on native PAGE without deproteinisation. DinG bound lssDNA with an apparent binding constant (K_app_) of <0.08 nM and efficiently degraded the substrate; however, traces of a high molecular mass complex were retained in the well (w) (Supplementary Figure S4A, lane 2). DinG D10A E12A, which does not degrade the substrate, binds the substrate with ~37-fold lower affinity than wt DinG. DinG D10A E12A formed a single protein-lssDNA complex (C1). At higher protein concentrations C2 complexes and complexes trapped in the well were also observed (Supplementary Figure S4A, lanes 11–19).

To prevent substrate degradation and test the metal dependence of binding, EMSAs were repeated in the presence of 2 mM EDTA with no added metal. DinG·EDTA and DinG K290A·EDTA bound γ^32^P-lssDNA_38_ with K_app_ of ~0.7 and ~1.1 nM, respectively. Thus, under these conditions, DinG·EDTA exhibited 10- to 15-fold lower affinity for lssDNA compared to when Mg^2+^ was added (Supplementary Figure S4A vs. Figure S4B lanes 2–10). At limiting concentrations, DinG or DinG K290A formed C1 complexes, whereas at above-stoichiometric concentrations they formed additional complexes: C2 complexes, and low-mobility complexes that were retained in the well (w) (Supplementary Figure S4B, lanes 9–10). Faint bands resulting from DNA degradation were still visible at high concentrations of DinG (lanes 6–10). By contrast, DinG D10A E12A·EDTA bound the substrate with ~two-fold lower affinity relative to its Mg^2+^-bound state (Supplementary Figure S4A, lanes 11–19 vs. S4B, 20–28).

### 2.8. DinG Appears to Advance in 4–5 nt Steps During 3′→5′ Degradation

**Figure 4:**
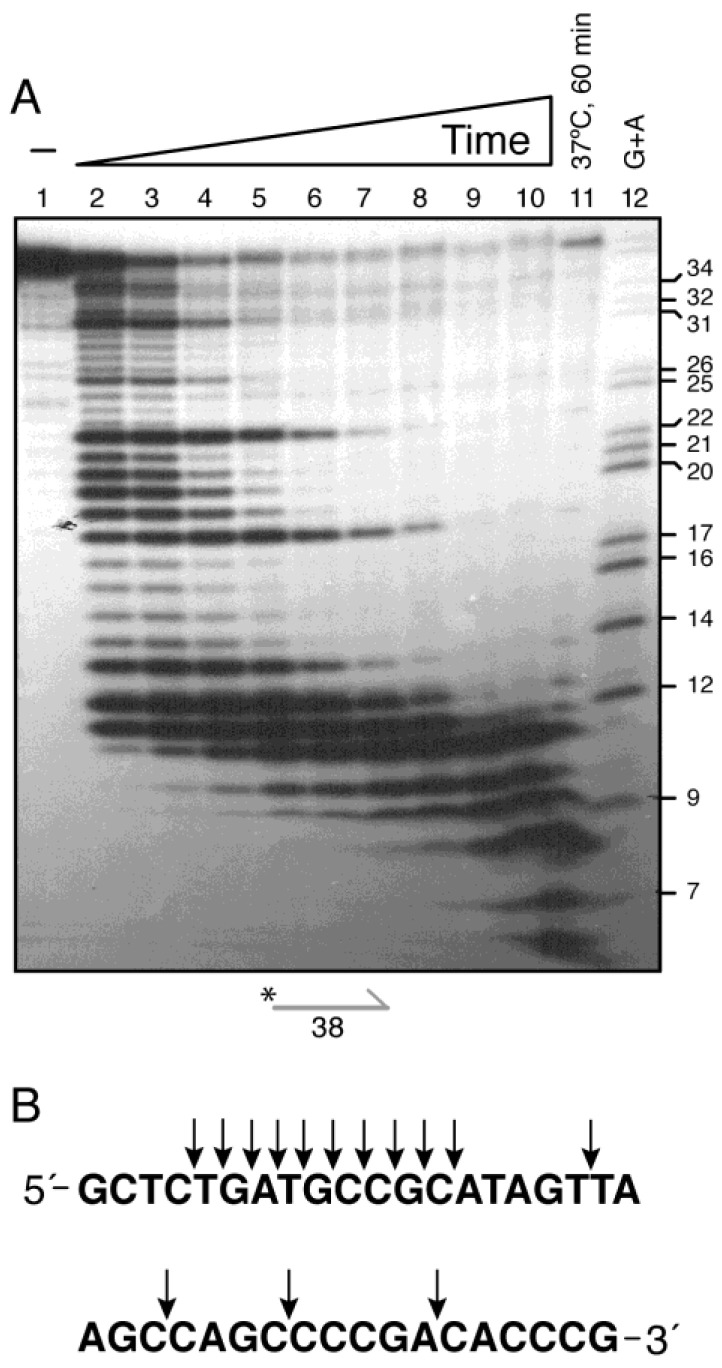
DinG bound to lssDNA undergoes different rounds of cleavage, in the 3′⟶5′ direction. (**A**) γ^32^P-lssDNA_38_ (5 nM in DNA molecules) was incubated with DinG (1 nM) for a variable time (0.5, 1, 2, 3.5, 5, 10, 15, 30, and 60 min) in buffer D at 22 °C (lanes 1–10) or for 60 min at 37 °C (lane 11). Reaction products were resolved by denaturing PAGE and visualised by autoradiography. “–”, no protein added; “G + A”, mobility marker, with the molecule lengths indicated in nt (lane 12); grey line, 5′-end labelled strand; solid line, DNA; “*”, labelled nucleotide; arrowhead, 3′-end; and number, length of each strand (in nt). All reactions were performed at least three times with consistent results, and a representative gel image is shown. (**B**) Arrows indicate cleavage sites on the lssDNA_38_ sequence, inferred from the accumulation of intermediates and products.

From the initial kinetics (Figure 1A), two models could explain how DinG degrades lssDNA under limiting enzyme conditions (0.1 monomer per DNA molecule) at 22 °C: (i) a conformational switch after early cleavage increases substrate affinity, allowing one-nucleotide stepping in low-processivity mode, with apparent pauses that yield 13–10 nt end-products; or (ii) the DinG exonuclease domain initially engages a 4 to 5 nt segment, effectively “steps” in 4–5 nt increments before rebinding downstream, until the DNA is reduced to 12 ± 1 nt in length. To test this, limiting DinG was incubated with γ^32^P-lssDNA_38_ (5 nM) over a 0.5–60 min time course at 22 °C. A DinG:ssDNA ratio of 0.2 monomers/lssDNA_38_ molecule—sufficient to bypass the “initial step” and simplify the analysis—was selected, and the reaction mixtures were separated under denaturing conditions (Figure 4).

After 30 s, over 50% of the substrate was degraded, yielding intermediates of 32, 27, 23, 18, and 14 to 12 nt (Figure 4A, lanes 2–3, and Figure 4B). These lengths correspond to sequential loss of 4–5 nt segments, consistent with a stepping mechanism. With extended incubation, the larger intermediates were further degraded, producing 13-9 nt products (Figure 4A, lanes 7–8). By 60 min—whether at 22 °C or 37 °C—only small end-products of 11-6 nt remained (Figure 4A, lanes 10–11). The discrete ladder of intermediates, spaced by ~4–5 nt (Figure 4B), indicates that DinG engages the 3′ terminus, cleaves one phosphate at a time, then repositions in ~4–5 nt increments before the next catalytic event. This stepping mechanism accounts for both the low overall processivity under limiting conditions and the characteristic pausing sites along ssDNA.

Extending this analysis, we examined two additional lssDNA substrates—a complementary 38 nt γ^32^P-lssDNA_38c_ and the 3′-Biotin γ^32^P-lssDNA_45_ (Figure 3A)—under varied protein concentrations and reaction times. Denaturing gels revealed recurring intermediates spaced by ~4–5 nt: (i) 3′-Biotin γ^32^P-lssDNA_45_ yielded fragments of (42), 39, 36-35, 32, 30, 28, 27, 24, 21-20, 16, and 15-6 nt (Supplementary Figure S5A, lanes 3–11); (ii) the γ^32^P-lssDNA_38c_ substrate produced bands at 33, 28, 24, 20-19 nt and final products of 12-5 nt (Supplementary Figure S5B, lanes 3–9). Analysis of the sequences at pause sites showed no enrichment for specific motifs or dinucleotides (Figure 4B and Supplementary Figure S5), indicating that pausing reflects an intrinsic 4–5 nt stepping mechanism rather than sequence-directed cleavage.

Based on kinetic and structural insights, we propose that DinG employs a “burst-and-anchor” mechanism: (i) the DinG*_Eco_* HD2 subdomain engages 4–5 nt of ssDNA [6], while the HD2 subdomain of DinG engages ~7 nt, anchoring the enzyme, with ~4 nt occupying the exonuclease active site along with two divalent cations, as observed in the DinG*_Sau_*-poly(dT_22_) structure [11]; (ii) limiting DinG concentrations cleave 1 nt at a time from the 3′-end until the ssDNA segment is too short to allow stable enzyme–substrate interactions (~12 nt), using a burst mechanism similar to that proposed for the ExoI*_Eco_* exonuclease [28]; (iii) after each cleavage event, the energy released from phosphodiester bond hydrolysis drives a rapid repositioning (“burst”) of the exonuclease domain 4 to 5 nt toward the new 3′ end, producing the discrete ladder of intermediates and potentially facilitating enzyme translocation along the lssDNA; and (iv) once the remaining ssDNA segment falls below ~12 nt, stoichiometric DinG or longer incubation times are required to generate 6 ± 1 nt end-products.

### 2.9. DinG Resects Fork DNA Via 3′-Tail Degradation

Previous studies have shown that: (i) DinG*_Sau_*, in the presence of 1 mM ATP, fails to unwind or degrade unreplicated fork structures over 60 min at 37 °C [10]; and (ii) saturating DinG*_Sau_* D10A, in the presence of 1–2 mM ATP, unwinds but does not degrade unreplicated forks within 20 min at 30 °C [11]. These contrasting behaviours prompted us to ask whether *B. subtilis* DinG couples ATP hydrolysis to mechanical fork unwinding or instead degrades the 3′-tail at stalled forks.

To investigate this, increasing concentrations of DinG were incubated with DNA substrates mimicking stalled forks: (i) a 3′-fork DNA structure, mimicking a replication fork with a fully synthesised leading strand and no nascent lagging strand (barrier in the lagging-strand); and (ii) a 5′-fork DNA structure, mimicking a replication fork with a fully synthesised lagging strand and no nascent leading strand (barrier in the leading strand). The γ^32^P-3′-fork or γ^32^P-5′-fork DNA substrates (0.5 nM, in DNA molecules) were incubated with increasing concentrations of DinG (0.15 to 25 nM) and fixed ATP (2.5 mM) for 10 min at 37 °C in buffer D. The reaction mixtures were then deproteinised and separated by native PAGE.

**Figure 5:**
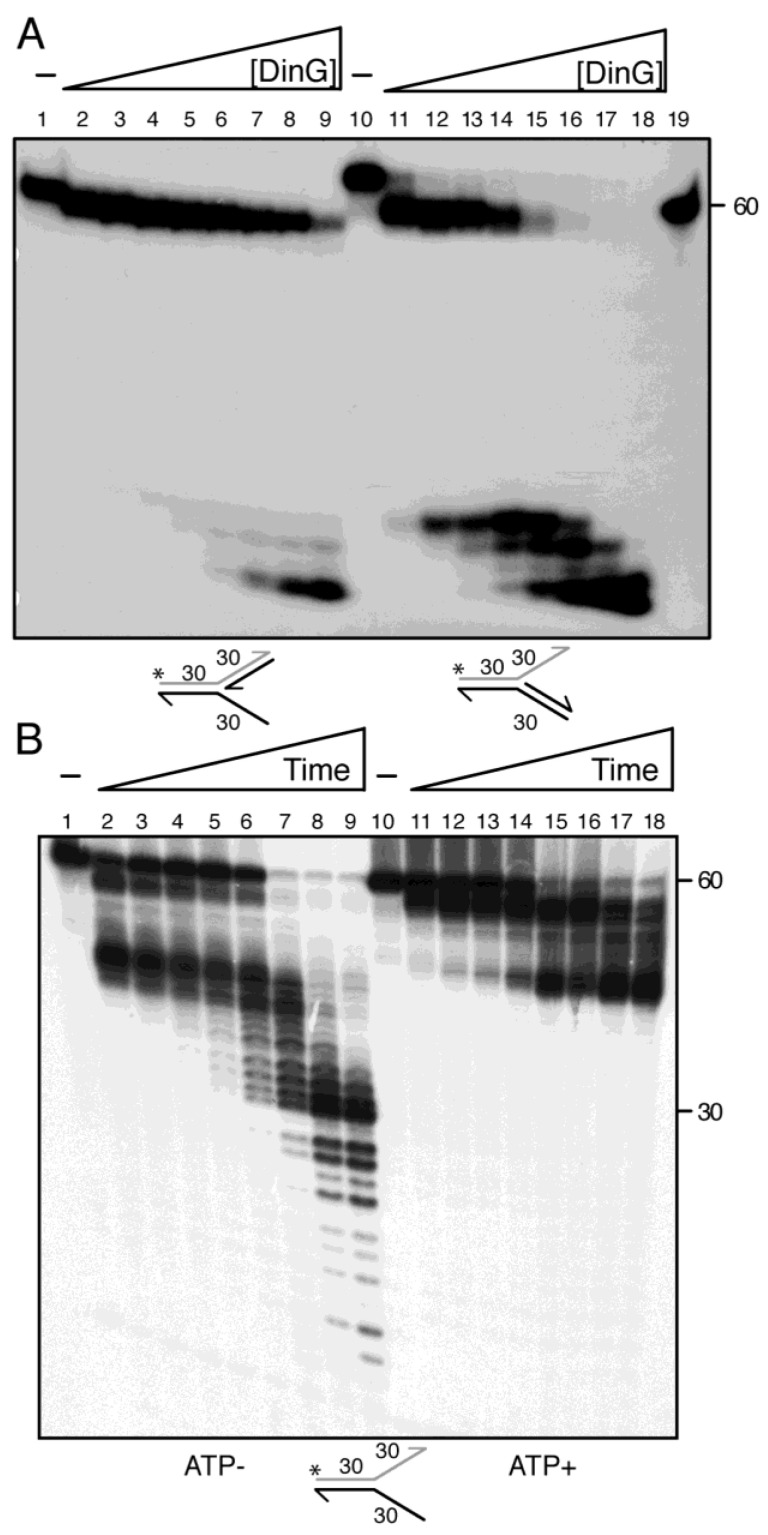
DinG degrades rather than unwinds fork DNA. (**A**) γ^32^P-3′-fork DNA (lanes 1–9) or γ^32^P-5′-fork DNA (lanes 10–18) (0.5 nM in DNA molecules) was incubated with increasing concentrations of DinG (0.15–25 nM) for 10 min at 37 °C in buffer D containing 2.5 mM ATP. Reaction mixtures were resolved by native PAGE. (**B**) γ^32^P-fork DNA (5 nM in DNA molecules) was incubated with DinG (0.5 nM) for a variable time (0.5–60 min) at 22 °C in buffer D, either lacking (lanes 1–9) or containing 2.5 mM ATP (lanes 10–18). The reaction products were then separated under denaturing conditions. “–”, no protein added; grey line, 5′-end labelled strand; solid line, DNA; “*”, labelled nucleotide; arrowhead, 3′-end; and number, length of each strand (in nt). All reactions were repeated at least three times with consistent results, and representative gel images are shown.

The 3′-fork DNA is a substrate for a helicase with 5′→3′ directionality, but not for an exonuclease with 3′→5′ polarity. Under limiting DinG·ATP concentrations (0.15–1 nM), the enzyme only slightly changed the mobility of the substrate (Figure 5A, lanes 2–3), indicating weak engagement. At saturating DinG·ATP concentrations (6–25 nM), the labelled strand was partially degraded, producing low mobility products (Figure 5A, lanes 7–9). Two non-exclusive mechanisms could explain this pattern: (i) DinG binds the 5′-tail, unwinds the unlabelled lagging strand, and then resects the resulting 5′-overhang—which is intrinsically a poorly exonuclease substrate requiring high enzyme levels for detectable trimming; and/or (ii) DinG directly attacks the nascent leading-strand arm, converting the substrate in an unreplicated fork that is then degraded from its free 3′ end. Either or both pathways may operate, reflecting DinG’s dual helicase–nuclease potential.

The 5′-fork DNA is a substrate for an exonuclease with 3′→5′ polarity, but not for a helicase with 5′→3′ polarity. Limiting DinG·ATP bound the 3′-tail and trimmed only a few nucleotides without disrupting the duplex junction (Figure 5A, lanes 11). At sub- to stoichiometric DinG·ATP levels, the junction was disrupted and the labelled template leading strand degraded (Figure 5A, lanes 11 vs. 12–13). With saturating DinG·ATP (6 DinG·ATP/5′-fork DNA molecule), the labelled strand was degraded to high mobility products, rather than being unwound the template leading strand (Figure 5A, lane 16–18). These results suggest the following: (i) ATP binding and hydrolysis do not supress the exonuclease activity of DinG, even on structured fork substrates; (ii) DinG lacks robust 5′→3′ helicase activity under these conditions; and (iii) DinG specialises in degrading recessed 3′-overhangs at stalled forks, consistent with a role in processing nascent strands or trimming R-loop-associated flaps.

### 2.10. DinG and DinG·ATP Differentially Degrade Unreplicated Forked DNA

To further probe whether DinG shows helicase activity, we used a simplified DNA substrate mimicking an unreplicated fork and analysed its degradation by DinG in the presence or absence of ATP. The unreplicated fork DNA substrate was incubated with substoichiometric concentrations of DinG or DinG·ATP over a time course ranging from 0.5 to 60 min at 22 °C in buffer D, and reaction products were analysed under denaturing conditions (Figure 5B).

In the absence of ATP, the 3′-tail of the unreplicated γ^32^P-fork DNA substrate was partially degraded within 30 s, yielding low mobility intermediates. After 5 min, these intermediates were further trimmed (Figure 5B, lanes 2–3 vs. 6). Prolonged incubation (7.5 to 15 min) led to partial destabilisation of the duplex region, producing smaller intermediates (Figure 5B, lanes 7–9). This suggests that DinG may use the chemical energy released from phosphodiester bond hydrolysis to drive limited translocation and progressive 3′-tail degradation, ultimately destabilising the duplex junction (Figure 5B, lanes 2–9 vs. 12–18).

A different outcome was observed when ATP (2.5 mM) was present. DinG·ATP cleaved ~50% of the 3′-tail within 30 s, yielding low mobility intermediates. After 2 min, degradation appeared to plateau, with accumulation of shorter low mobility species (Figure 5B, lane 11 vs. 14–18), indicating that DinG·ATP stalls upstream of the junction. These results suggest the following: (i) apo DinG progressively destabilises the forked structure, consistent with exonucleolytic progression across the junction (Figure 5B, lanes 6–9); (ii) ATP does not inhibit DinG exonuclease activity on forked substrates; and (iii) DinG·ATP preferentially degrades the 3′-tail and stalls upstream of the junction rather that unwinding the substrate to generate a lssDNA product susceptible to further degradation (Figure 5B, 15–18).

### 2.11. DinG Degrades Recessed DNA in 5′-Overhang Substrates

**Figure 6:**
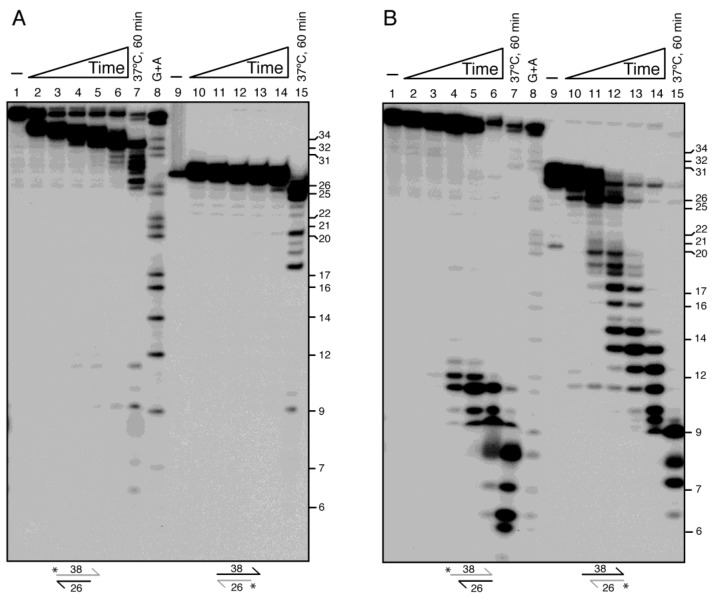
DinG differentially degrades DNA substrates with 3′- and 5′-overhangs. (**A**) 3′-overhang γ^32^P-duplex DNA or (**B**) 5′-overhang γ^32^P-duplex DNA (5 nM in DNA molecules), where either the long or short strand was radiolabelled, was incubated with DinG (1 nM) for a variable time (0.5, 1, 2, 3.5, 5, 10, 15, 30, and 60 min) at 22 °C, or for 60 min at 37 °C (lanes 7 and 15), in buffer D. Reaction products were resolved by denaturing PAGE. “–”, no protein added; “G + A”, mobility marker, with the molecule lengths indicated in nt (lane 8); grey line, 5′-end labelled strand; solid line, DNA; “*”, labelled nucleotide; arrowhead, 3′-end; and number, length of each strand (in nt). All reactions were repeated at least three times with consistent results, and representative gel images are shown.

To determine the fate of the unlabelled nascent leading strand in the 3′-fork substrate (Figure 5A, lanes 2–5) and to test whether DinG can degrade DNA ends within a duplex context, we employed duplex DNA substrates bearing either 3′ or 5′ overhangs. These substrates were labelled on one strand or the other and incubated for variable time at 22 °C to slow degradation kinetics and facilitate analysis (Figure 6). Specifically, γ^32^P-labelled 38 or 26 nt single-stranded DNA (γ^32^P-lssDNA_38_ or γ^32^P-lssDNA_26_) was hybridised with complementary unlabelled 26 or 38 nt strands, respectively. This produced 3′- or 5′-overhang γ^32^P-dsDNA substrates (Supplementary Figure S3 and Table S2), enabling us to assess whether DinG interacts asymmetrically with either strand, and explore DinG’s interaction with recessed ends mimicking forked replication intermediates.

Limiting DinG concentrations degraded >50% of the 3′-tailed γ^32^P-dsDNA_38_ substrate within 1 min, yielding a 34 ± 1 nt intermediate (Figure 6A, lanes 2–3). Prolonged incubation modestly trimmed these intermediates, halting 6 ± 1 nt upstream of the duplex junction (Figure 6A, lane 6). However, when incubated at 37 °C, DinG further degraded the substrate—likely through partial duplex destabilisation or spontaneous strand melting, (Figure 6A, lane 7). In contrast, when the complementary strand was labelled (γ^32^P-dsDNA_26_), minimal degradation occurred over 60 min at 22 °C from the blunt 3′-end (Figure 6A, lane 14), but partial digestion and a modest accumulation of a 9 nt product emerged at 37 °C.

When the 5′-overhang γ^32^P-dsDNA_38_ substrate was incubated with limiting DinG, no appreciable degradation of the labelled strand from the blunt 3′-end was observed during the first 5 min. By 15 min, partial trimming yielded 11 ± 1 nt intermediates, and by 60 min >70% of the labelled strand was converted into 11 ± 2 nt products at 22 °C or 7 ± 2 nt products at 37 °C (Figure 6B, lanes 6 vs. 7). In contrast, when the bottom strand was labelled, DinG degraded >95% of the recessed γ^32^P-strand of the substrate within 2 min, producing intermediates ranging from 26 to 13 nt (Figure 6B, lane 12). After 60 min, final products of 11 ± 2 nt (22 °C) and 7 ± 1 nt (37 °C) accumulated (Figure 6B, lanes 12–14 vs. 15).

From the data in Figure 6, it is likely that: (i) DinG engages and trims the 3′ overhang, halting 5 ± 1 nt before the duplex junction at 22 °C (Figure 6A, lanes 2–6); (ii) in the presence of a 3′-tail—which may titrate limiting DinG—the enzyme does not efficiently target blunt-ended duplex DNA at 22 °C, but can partially destabilise the duplex region and degrade the lssDNA at 37 °C; (iii) when bound to the ssDNA of the 5′-overhang substrate, DinG disrupts base pairing and degrades the recessed bottom strand from its 3′-end, thereby exposing the 3′-end of the top labelled strand (γ^32^P-dsDNA_38_) for subsequent degradation (Figure 6B lanes 2–6); and (iv) apo DinG bound to 5′-overhangs destabilises the substrate at the duplex interface, enabling degradation of the 3′-labelled strand, using the energy released from phosphodiester bond hydrolysis to promote translocation and subsequent degradation of the resected 3′ends. This mechanism may also underlie the activity observed when DinG·ATP was incubated with 3′-fork DNA (Figure 5, lanes 2–9). Similarly, DinG*_Sau_* degrades the recessed strand in 5′-overhang substrates [10].

### 2.12. DinG Degrades the RNA Strand of RNA–DNA Hybrids

In previous experiments, a notable discrepancy emerged among DinG homologues from the Bacillales order. In the presence of a large excess of protein (∼50 DinG*_Sau_* monomers/lssRNA molecule), DinG*_Sau_* preferentially degraded lssRNA at 37 °C [10]. By contrast, an excess of DinG (∼10 DinG monomers/lssRNA molecule) failed to degrade lssRNA at 22 °C (Figure 1C, lanes 2–9). These divergent outcomes may result from the spontaneous self-annealing of lssRNA, which could hinder access to its 3′-end, or they may reflect substrate-specific preference among Bacillota DinG orthologues.

To test these possibilities, γ^32^P-lssRNA_38_ (indicated by a dashed lane) was annealed with a complementary unlabelled lssDNA_26_ to generate structured 3′- or 5′-overhang γ^32^P-RNA–DNA hybrids (Supplementary Figure S3). These substrates (5 nM in DNA molecules) were incubated with increasing concentrations of DinG (0.3–24 nM and 100 nM) in buffer D for 10 min at 37 °C, either in the absence or presence of 2.5 mM ATP. The reaction products were then separated under denaturing conditions. This experimental design allowed us to distinguish nonspecific RNA degradation from substrate recognition, thereby providing insight into how DinG engages RNA–DNA hybrids depending on overhang polarity and reaction conditions.

**Figure 7:**
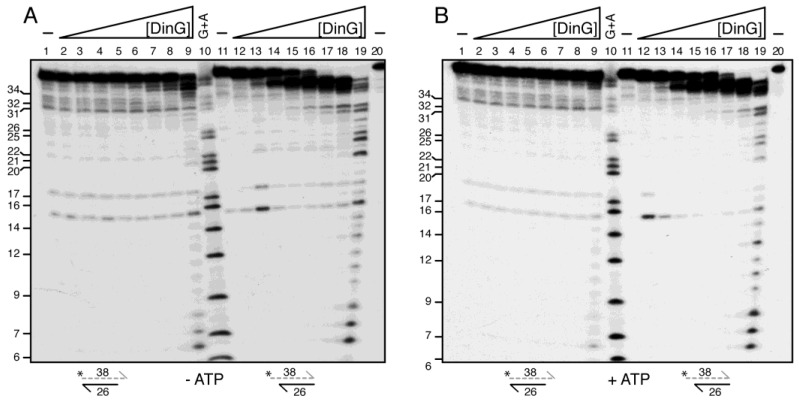
DinG degrades the RNA strand of RNA–DNA hybrids. (**A**,**B**) 3′-overhang γ^32^P-RNA-DNA (lanes 2–9) or 5′-overhang γ^32^P-RNA-DNA (lanes 12–19) (5 nM in DNA molecules) was incubated with increasing concentrations of DinG (0.3–24 and 100 nM) for 10 min at 37 °C in buffer D either lacking (**A**) or containing 2.5 mM ATP (**B**). “–”, no protein added; “*”, labelled nucleotide; arrowhead, 3′-end; grey line, 5′-end labelled strand; solid line, DNA; dashed line, RNA; “G + A”, mobility marker, with the molecule lengths indicated in nt (lane 10); and number, length of each strand (in nt). All reactions were performed at least three times with consistent results, and representative gel images are shown.

As expected, limiting to just above stoichiometric concentrations of DinG did not degrade the 3′-tailed RNA strand of the 3′-overhang γ^32^P-RNA–DNA hybrid substrate (Figure 7A, lanes 2–5), confirming that DinG does not degrade lssRNA. At approximate stoichiometry (1.2 DinG/3′-overhang RNA-DNA molecule), DinG trimmed a few nucleotides from the 3′-end of the labelled RNA strand (Figure 7A, lane 6). A large excess of DinG (20 DinG/hybrid molecule) was required for more extensive trimming, producing 36 to 34 nt intermediates and, with reduced efficiency, 8 to 6 nt end-products (Figure 7A, lane 9). Similar results were obtained in the presence of ATP, although overall degradation was less efficient (Figure 7B, lanes 7–9), likely due to reduced substrate affinity (see above).

When incubated with the 5′-overhang γ^32^P-RNA–DNA hybrid substrate, limiting DinG cleaved ~50% of the labelled RNA strand from its 3′-blunt end, yielding a 34 ± 2 nt γ^32^P-lssRNA intermediate (Figure 7A, lane 14). At a moderate enzyme-to-substrate ratio (2.4 DinG/RNA–DNA hybrid molecule), degradation proceeded further, although DinG·ATP showed reduced efficiency (Figure 7A,B, lane 17). At high DinG excess (20 DinG/hybrid substrate), degradation progressed further to yield a ladder of intermediates and distinct 8 to 6 nt end-products (Figure 7A, lane 19). Similar patterns were observed in the presence of ATP, though with lower overall activity (Figure 7B, lane 19).

At least two possible mechanisms could explain DinG-mediated RNA degradation: (a) DinG recognises hybrid structures—particularly the interface between ssRNA and duplex regions; or (b) DinG bound to the blunt end of the 5′-overhang hydrid destabilises the duplex, enabling partial degradation of the γ^32^P-RNA strand. We consider it unlikely that DinG first degrades the ssDNA strand and then the γ^32^P-RNA strand, because DinG cannot degrade lssRNA (Figure 1C, lanes 2–9). To test this, we examined whether DinG could degrade blunt-ended RNA–DNA hybrids. A γ^32^P-lssRNA_38_ strand was annealed to a complementary unlabelled lssDNA_38_ strand (Supplementary Figure S3) to form a blunt-ended RNA–DNA hybrid. This substrate was incubated with increasing concentrations of DinG for 15 min at 37 °C in buffer D, either with or without 2.5 mM ATP (Supplementary Figure S6). In the absence of a 3′-tail, both DinG and DinG·ATP degraded the blunt-ended RNA–DNA hybrid, although ATP again reduced activity (Supplementary Figure S6). These results suggest the following: (i) DinG selectively recognises hybrid substrates and degrades the γ^32^P-RNA strand from the 3′-end, and (ii) DinG utilises the chemical energy released from RNA strand hydrolysis to fuel limited mechanical movement, rather than relying on ATP hydrolysis for translocation.

### 2.13. DinG Partially Degrades RNA–DNA hybrids Present on Genomic DNA

**Figure 8:**
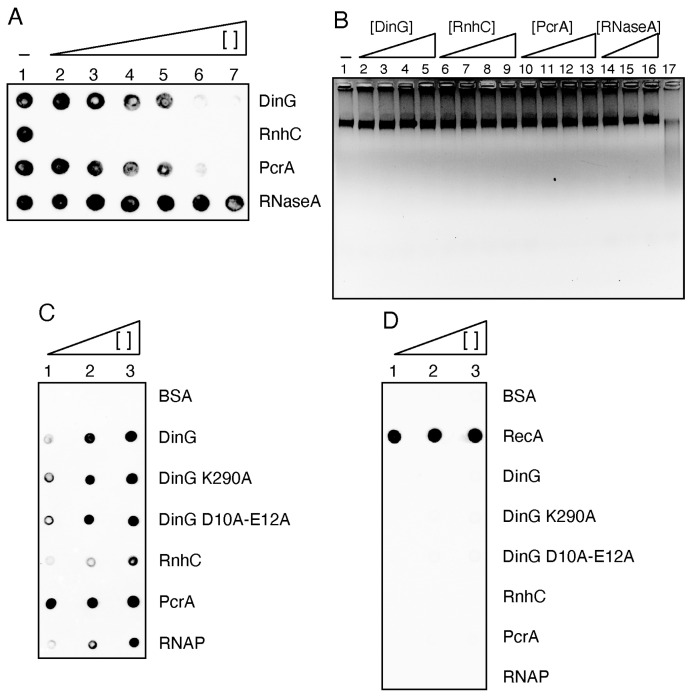
DinG partially degrades RNA–DNA hybrids. (**A**) Purified gDNA from exponentially grown Δ*rnhC* cells, transiently exposed to MMS-induced lesions (20 mM for 15 min), was incubated with increasing concentrations of DinG, RnhC, PcrA, or RNaseA (30–1000 nM) for 15 min at 37 °C in buffer E with or without 5 mM ATP. Reaction mixtures were spotted onto positively charged Nylon membranes. R-loops were detected using S9.6 mAb. (**B**) The gDNA was incubated with increasing concentrations of DinG, RnhC, PcrA, or RNaseA (125–1000 nM) or a fixed concentration of DNase I (5 nM, lane 17) for 15 min at 37 °C in buffer E with or without 5 mM ATP. The integrity of gDNA was then assayed by agarose gel electrophoresis stained with ethidium bromide. “–”, no protein added. (**C**,**D**) RecA interacts with DinG, RnhC, PcrA, and RNAP. Increasing amounts of DinG, DinG D10A E12A, DinG K290, RnhC, PcrA, RNAP, BSA, or RecA (in (**D**)) (75–300 ng) were spotted onto nitrocellulose membranes. In panel (**C**), membranes were incubated with a fixed concentration of RecA (200 ng·ml^−1^) and washed. In both panels, polyclonal anti-RecA antibodies were used to detect the retained RecA signal. All reactions were performed at least three times with consistent results, and representative membranes are shown.

To investigate whether DinG can degrade RNA–DNA hybrids formed in vivo, genomic DNA (gDNA) was purified from a Δ*rnhC* strain exposed to 20 mM MMS for 15 min, which accumulates R-loops [20], and tested in vitro with purified proteins (Figure 8A). A bona fide R-loop on gDNA can be defined by four criteria: (a) resistance to pancreatic ribonuclease A (RNase A), which efficiently degrades ssRNA or dsRNA but not RNA–DNA hybrids [29]; (b) sensitivity to RnhC, which specifically degrades the RNA strand of RNA–DNA hybrids [26]; (c) selective recognition by the S9.6 monoclonal antibody (mAb), which binds RNA–DNA hybrids with ~20-fold higher affinity than dsRNA and does not interact with ssDNA or dsDNA [30,31]; and (d) unwinding of the RNA strand by the PcrA helicase in the presence of ATP [27].

A fixed amount of gDNA (650 μM in nt molecules) was incubated with increasing concentrations of each control protein. Reaction products were spotted onto a membrane, which was subsequently probed with S9.6 mAb. In the mock reaction (no protein added), a strong signal was detected (Figure 8A, lane 1). Treatment with increasing concentrations of RNase A in buffer E did not significantly reduce this signal. By contrast, very low concentrations of RnhC (1 RnhC monomer/>21,000 bp) completely abolished the S9.6 signal (Figure 8A, lanes 2–7 vs. lane 1) without reducing total gDNA levels (Figure 8B, lanes 6–9). Furthermore, PcrA·ATP (30–1000 nM) removed >95% of the detected signal (Figure 8A, lane 5 vs. 6–7).

Under limiting conditions (1 DinG monomer/5200 bp), DinG removed ~50% of the S9.6 mAb signal within 15 min at 37 °C. At higher concentrations (500 nM), DinG abolished >95% of the signal (Figure 8A, lane 4 vs. 6). Importantly, total gDNA levels remained unchanged in the presence of DinG, whereas gDNA was degraded by DNase I (Figure 8B, lanes 2–5 vs. 17). These results indicate that DinG selectively degrades RNA–DNA hybrids without significantly affecting bulk gDNA.

We propose that DinG, acting mechanistically as a distributive exoribonuclease on 5′-overhang DNA, may facilitate RnhC activity as an endoribonuclease on both 5′-overhang and blunt-ended RNA–DNA hybrids [32]. These findings are consistent with previous reports identifying RnhC as the primary R-loop degrading enzyme [20,26].

### 2.14. DinG Interacts with RecA, Which Provides a Platform to Overcome Replication Stress

Single-molecule imaging in live unstressed cells has revealed several key features of replisome and RecA dynamics: (i) the replisome pauses at highly transcribed regions such as the *rrn* loci [15] and undergoes spontaneous disassembly ~five times per cell cycle, followed by reassembly once the barrier is overcome [16]; (ii) a stalled replisome is required for spontaneous RecA foci on the nucleoid in ~15% of total *lexA*^+^ and *lexA*(Ind^−^) cells, and in >85% of these cases the foci colocalise with DnaX [17]; (iii) RecA foci are spontaneously enriched at *rrn* loci or at engineered RTCs, as shown by ChIP-seq [19]; and (iv) upon exposure to limiting (1 J/m^2^) or higher (40 J/m^2^), which trigger the SOS response, UV doses, ~85% and ~97% of total cells, respectively, display RecA foci, with ~90% colocalisation with DnaX [17]. To determine whether RecA interacts with proteins involved in R-loop resolution, we performed immuno-dot blot assays with purified proteins. Specifically, we tested RecA interaction with DinG, its mutant variants (DinG D10A E12A and DinG K290A), RnhC, PcrA, and the RNAP complex. PcrA was included as a positive control, since it is known to interact with RecA [33], while BSA served as a negative control.

Each protein (75–300 ng) was serially diluted and spotted onto a nitrocellulose membrane. The membrane was incubated with RecA (200 ng·mL^−1^), extensively washed, and probed with polyclonal anti-RecA antibodies to detect retained RecA. As expected, no signal was observed for BSA (300 ng) (Figure 8C, lane 3), while PcrA (75 ng) retained RecA (Figure 8C, lane 1), confirming assay specificity. DinG and both mutant variants (150 ng each) also interacted with RecA (Figure 8C, lanes 2–3). A higher amount (300 ng) of RnhC was required to detect the interaction (Figure 8C, lane 3). The RNAP complex (150 nM) also retained RecA (Figure 8C, lane 2). To exclude antibody cross-reactivity, RecA and each test protein (75–300 ng) were separately spotted, the membrane was washed extensively and probed with anti-RecA antibodies (Figure 8D). Only RecA produced a detectable signal, confirming that the observed signals belong to RecA.

### 2.15. DinG-mGold Molecules Are Recruited to DnaX-CFP upon Endogenous or Environmental Threats

**Figure 9:**
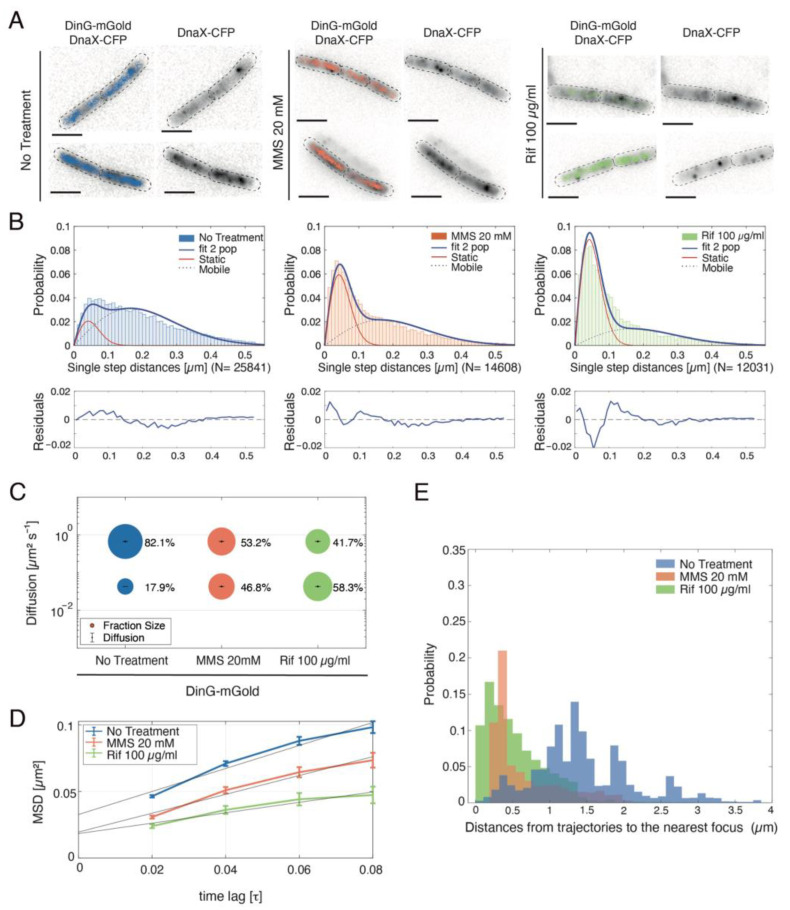
Single-molecule tracking of DinG-mGold. (**A**) Live-cell analysis of treated (MMS (red) or Rif (green)) or untreated (blue) cells. Representative images of DnaX-CFP localisation and overlaid tracks of DinG-mGold in *B. subtilis*, either untreated or after treatment. (**B**) Jump distance analysis shows the probability of displacements: solid red lines represent the static subpopulations, dashed blue lines the mobile fraction, and solid blue lines the sum of both subpopulations. (**C**) Bubble plots comparing fraction sizes (bubble size) and diffusion constants (y-axis) under different conditions. A simultaneous fit was applied, to show that changes in molecule dynamics are reflected in changes in populations sizes rather than in different diffusion constants. Step-size distributions reveal two populations for each condition: a mobile (upper circles) and a static (lower circles) fraction. (**D**) Mean squared displacement analyses assuming a single population model. (**E**) Histogram showing the distances of DinG-mGold trajectories from DnaX-CFP in 50 cells under each condition.

Having characterised the enzymatic activities of DinG in vitro, we next investigated its role in vivo. First, we analysed its localisation within the cell by expressing a DinG-mGold fusion from the native *dinG* locus (Figure 9). The fusion protein retained full functionality, as shown by identical survival rates of the DinG-mGold and wt strain following acute exposure to 20 mM MMS for 15 min.

We used real-time single-molecule imaging to track DinG dynamics during exponential growth. DinG dynamics were visualised using 20 ms stream acquisitions, and tracks consisting of fewer than five steps were discarded to minimise bias from short tracking events. An example of DinG-mGold trajectories in four cells is shown in Figure 9A, where the tracks are confined to the central regions of the cells—a hallmark of proteins associated with replisomes. We observed that, during vegetative growth, DinG-mGold moves within the bacterial cell; however, in a small fraction of unstressed cells, it formed spontaneous foci at the nucleoid during vegetative growth. To characterise the dynamic behaviour of DinG, we performed mean squared displacement (MSD) analyses. The results are displayed in Figure 9B as jump distance distributions, in which the probability of different DinG squared displacement lengths is plotted. By assuming two populations with distinct average diffusion constants, we were able to effectively describe the observed step-distribution, and fitting the data with two Rayleigh distributions yielded an R^2^ value of 0.99. These data suggest the presence of a mobile and freely diffusing population of DinG (comprising 82% of total DinG foci), and a static (18% DinG foci), likely DNA-bound fraction (Figure 9C,D, Table 1). The diffusion constant of 0.043 µm^2^ s^−1^ determined for the static DinG population is comparable to that of proteins tightly bound to DNA [34,35]. Thus, DinG-mGold moves within the central areas of unstressed cells and forms spontaneous foci at the nucleoid during vegetative growth in a small fraction of them.

To determine whether DinG colocalises with replication forks, DnaX-CFP foci were analysed concomitantly. Tracks colocalised with—but were not restricted to—DnaX-CFP foci (Figure 9A). Similarly, the DinG_Eco_ DNA helicase may also localise to and interact with replisome functions, as it contains a conserved pentanucleotide motif (QLDLF) predicted to interact with the DnaN-sliding clamp. However, the functional significance of this clamp-binding motif remains undetermined.

To investigate whether DinG-mGold dynamics are altered by treatment with chemicals affecting replisome progression, cells were exposed to MMS, which halts both DNAP and RNAP elongation [36], or to the transcription inhibitor Rifampicin (Rif). Rif targets the RpoB subunit in both close and open RNAP-DNA complexes to block transcription initiation, but it neither binds to nor affects the progression of the RNAP elongation complex [37]. Rif also prevents the localisation of Topo I at RNAP-promoter complexes, indirectly increasing negative super helicity and R-loop formation at elongating RNAP [38,39,40].

As shown in Figure 9D, both MMS and Rif profoundly impacted DinG molecule dynamics, which were strongly decreased. Furthermore, this slowdown of molecules is based on a strong increase in slow-mobile molecules and a concomitant decrease in the fast-mobile fraction (Figure 9B,C). Concretely, our analyses reveal that during exponential growth: (i) ~18% of DinG molecules exhibit static motion, which we interpret as being bound to their substrate; (ii) after the induction of DNA damage by MMS treatment, this percentage rises to ~47% (Figure 9C), and (iii) blocking transcription initiation further augments the static state of DinG to even ~58% (Figure 9C).

To investigate whether static DinG molecules are bound to (likely stalled) replication forks, we measured the distance of DinG tracks to the nearest replication fork, visualised by DnaX-CFP. In unstressed, exponentially growing cells, DinG-mGold tracks centred around DnaX-CFP foci were observed (left panel, in Figure 9A), indicating occasional recruitment of DinG to replication forks, possibly due to spontaneously arising barriers. However, as shown in Figure 9E, only a small fraction of these DinG tracks is in proximity to DnaX-CFP foci, suggesting that a limited portion of the ~18% of static tracks represent replication fork-associated DinG molecules. In contrast, the presence of MMS and even more so with Rif, resulted in clear shift of DinG molecules towards replication forks (Figure 9E). A substantial majority of DinG tracks now exhibited proximity to DnaX-CFP foci (notice that the localisation error is 250 nm due to the diffraction limit of DnaX-CFP signals, while DinG-mGold signals have a precision below 50 nm). Therefore, ~47% to ~58% of static DinG molecules observed after MMS or Rif treatment appear to be bound to replication forks. Although we cannot quantify the exact degree of fork-recruitment, it is reasonable to conclude that treatment with MMS or Rif leads to a major shift in the dynamics of DinG molecules from a freely diffusive into a replication fork-engaged state. This observation suggests that DNA damage or enhanced RTCs formation leads to a pronounced recruitment of DinG to the stalled replication machinery.

### 2.16. Absence of DinG Does Not Affect End Resection

The Xon*_Eco_* (also known as ExoI*_Eco_*) 3′⟶5′ exonuclease enzyme is crucial for removing “dirty” DNA ends during basal end-resection [41]. *B. subtilis* lacks an ExoA-like protein, although the DinG exonuclease domains share significant sequence identity with Xon*_Eco_* (Supplementary Figure S1A). Notably, DinG is enriched on dsDNA fragments containing ribonucleoside monophosphates and abasic sites [42], lesions that persist following incomplete repair of MMS-induced damage [43]. To test whether DinG contributes to the removal of such “dirty” DNA ends, a null *dinG* (Δ*dinG*) strain was grown to an OD_560_ = 0.4 at 37 °C with shaking and plated on LB agar supplemented with MMS or H_2_O_2_, which induce alkylating or oxidative DNA damage, respectively (Supplementary Figure S7). The survival of Δ*dinG* cells was comparable to that of the wt, indicating that the loss of DinG does not increase sensitivity to these agents.

We next hypothesised that DinG might function as an accessory factor for canonical end-resection enzymes. To test this, Δ*dinG* was introduced into Δ*addAB*, Δ*recJ*, Δ*recQ*, and Δ*recS bona fide* end-resection mutants. As detailed in Supplementary Section SA, the Δ*dinG* mutation neither altered recombination phenotypes (natural chromosomal and plasmid transformation) nor increased sensitivity to DNA-damaging agents in these mutant backgrounds (Supplementary Figure S7A–C). Taken together, these results indicate that DinG does not play a major role in basal end resection, and that other 3′⟶5′ exonuclease can process these 3′-tails (e.g., PNPase) [44]. Alternatively, DinG function may overlap with that of its paralogue YpvA or with that of other specialised proteins that operate during replication stress.

### 2.17. DinG and YpvA Differentially Contribute to Cell Survival Following DNA Damage

To explore the potential interplay of the DinG and YpvA paralogues, the Δ*ypvA* mutation was moved to Δ*dinG* cells. Strains were grown to OD_560_ = 0.4 at 37 °C with shaking and plated on LB agar supplemented with increasing concentrations of MMS. Interestingly, the Δ*ypvA* mutation significantly increased survival and partially suppressed the sensitivity to alkylating lesions of wt or Δ*dinG* strains (Supplementary Figure S8A).

While this manuscript was in preparation, it was reported that DinG*_Sau_* inactivation causes moderate sensitivity to MMC, but not MMS, and it was proposed that the DNA helicase and exonuclease activities of DinG*_Sau_* contribute to removing DNA cross-links [11]. To test whether DinG, alone or in concert with YpvA, contributes to the removal of MMC-induced cross-links, the Δ*dinG*, Δ*ypvA*, Δ*dinG* Δ*ypvA,* and wt strains were exposed chronically to 180 nM MMC. Both wt and Δ*dinG* cells were highly sensitivity to MMC, whereas Δ*ypvA* and Δ*dinG* Δ*ypvA* strains were significantly more resistant (Supplementary Figure S8A). These results suggest the following: (i) the putative 5′⟶3′ helicase YpvA antagonises specialised repair pathway(s), such that it facilitates more efficient removal of intra- and inter-strand crosslinks; and (ii) the DinG 3′⟶5′ exonuclease poorly contributes, if at all, to the removal of such lesions.

To further dissect the in vivo roles of DinG and YpvA, we overexpressed wt *dinG*, the exonuclease-dead *dinG* D10A E12A, the ATPase-deficient *dinG* K290A, or wt *ypvA* from a plasmid-borne inducible promoter (Supplementary Section SB). Overexpression of wt DinG, DinG K290A, or YpvA significantly increased doubling time, reduced plating efficiency, and (for DinG) decreased colony size (Supplementary Figure S9A–E). In contrast, overexpression of DinG E10A D12A had no measurable effect on cell viability or growth rate, indicating that the toxicity of DinG depends on its exonuclease activity but not on ATP hydrolysis (Supplementary Figure S9A–E).

### 2.18. dinG Inactivation Does Not Increase the Sensitivity of ΔrnhC or ΔrecA Cells to DNA Damage

Recent genetic analyses have demonstrated the following: (i) *pcrA* is not epistatic to *dinG* or *rnhC* under replication fork-stalling damage such as MMS treatment [27,45]; (ii) *dinG* is not epistatic with *recD2* in response to MMS- or H_2_O_2_-induced DNA damage [46]; and (iii) the Δ*rnhC* mutation is synthetically lethal in a Δ*recA* background but not in Δ*dinG* or Δ*rnhB* strains [27]. To assess whether *dinG* genetically interacts with *recA*, *rnhB,* or *rnhC*, we introduced the Δ*dinG* mutation into Δ*rnhB*, Δ*rnhC,* and Δ*recA* strains. These single and double mutants were subjected to chronic MMS exposure, as alkylated DNA bases stall both DNAP and RNAP elongation [43,47]. The Δ*dinG* Δ*rnhB*, Δ*dinG* Δ*rnhC*, or Δ*dinG* Δ*recA* double mutants exhibited survival rates indistinguishable from those of their most sensitive single mutant parental strain (Supplementary Figure S8B,C).

### 2.19. Absence of DinG Reduces Mutagenesis

DinG shares sequence identity with the 3′⟶5′ proofreading domains of replicative PolC and DnaQ/ε*_Eco_* enzymes (Supplementary Figure S1B) and localises to replication forks in response to DNA damage (Figure 9). It can therefore be hypothesised that DinG could either act as a backup proofreading enzyme or assist TLS DNAPs by removing unpaired 3′-ssDNA flaps. To distinguish between these two mutually exclusive models, we measured spontaneous and MMS-induced mutation frequencies in a prophage-free strain [48].

The Δ*dinG* mutant exhibited a statistically significant (*p* < 0.01), two- to three-fold reduction in both spontaneous (0.8 × 10^−9^ ± 0.4 × 10^−9^) and MMS-induced (2.8 × 10^−9^ ± 0.4 × 10^−9^) mutation frequencies compared with the wt strain (2.1 × 10^−9^ ± 0.3 × 10^−9^ and 5.6 × 10^−9^ ± 0.8 × 10^−9^, respectively) (Supplementary Figure S10A). Since the absence of DinG confers an anti-mutator phenotype, we reasoned that: (i) DinG may decrease replication fidelity by promoting lesion bypass, allowing replication to resume; or (ii) the DinG 3′→5′ exonuclease activity may resect unannealed 3′ ends, thereby facilitating recruitment of TLS DNAPs for error-prone bypass of damaged template bases.

To explore this model, we examined genetic interactions between DinG and the three dispensable host-encoded TLS DNAPs PolA, PolY1, and PolY2, all of which lack 3′⟶5′ proofreading activity [49], and references therein. Previous work showed that Δ*polA* Δ*polY1* or Δ*polA* Δ*polY2* double mutants exhibit mutation rates similar to the Δ*polA* single mutant, indicating functional cooperation [49,50], although the contribution of the prophage SPβ-encoded TLS DNAP YolD-UvrX (belonging to the PolY2 clade) had not been ruled out. We therefore introduced the Δ*dinG* mutation in the Δ*polY1*, Δ*polY2*, and Δ*polA* strains lacking SPβ (Supplementary Table S1).

As expected, Δ*polY1* or Δ*polY2* single mutants had little effect on spontaneous mutation rates but significantly reduced damage-induced mutagenesis (Supplementary Figure S10A) [48]. Similar results were observed when YolD-UvrX was present [49,51]. In Δ*dinG* Δ*polY1* cells, both spontaneous and MMS-induced mutation frequencies were indistinguishable from those of Δ*dinG*. Differently, in Δ*dinG* Δ*polY2* cells, spontaneous mutagenesis resembled Δ*dinG*, while MMS-induced mutation rate resembled Δ*polY2* (Supplementary Figure S10A). Δ*polA* cells showed almost no spontaneous mutagenesis and only a small residual MMS-induced mutation frequency (Supplementary Figure S10A). Likewise, a small fraction of UV-induced mutagenesis occurred in Δ*polA* cells carrying SPβ [49]. The Δ*polA* Δ*dinG* double mutant phenocopied Δ*polA* in both mutation frequencies (Supplementary Figure S10A). Together, these results support a model in which DinG cooperates with PolA to promote error-prone lesion bypass, although its precise mechanism remains undefined.

Previous studies have reported the following: (i) PolA, PolY1, DinG, and the essential DnaN sliding clamp are constitutively expressed, whereas PolY2 and RecA are LexA-regulated [9]; (ii) PolA and DnaN assemble into ternary complexes with PolY1 or PolY2, forming bipartite TLS DNAPs see [49]; and (iii) RecA physically interacts with DinG (Figure 8C). Using bacterial two-hybrid assays with PolY1 and DnaN as positive controls, we found that PolA interacts with DinG, DnaN, PolY1, and RecA (Supplementary Figure S10B,C). Given that PolA and PolY1 form spontaneous and damage-induced foci at or near sites of replication [21,22], the interaction between PolA and DinG may be indirect. To determine whether ssDNA regions mediate these interactions, we tested PolY1 and PolY2 as probes. No interactions were observed between DinG and either PolY1 or PolY2 (Supplementary Figure S10B). These data reinforce the idea that DinG acts together with PolA during mutagenic lesion bypass. Future studies should determine how DinG and PolA co-ordinately modulate this error-prone DNA damage tolerance sub-pathway.

## 3. Conclusions

### DinG, as a Helpmate, Contributes to Mitigate Replication Stress, a Proposed Model

In this study, we combined genetic, cytological, and biochemical approaches to characterise the role of *B. subtilis* DinG in conjunction with other functions required to overcome replication stress. Our data support a working model in which DinG acts as an auxiliary “helpmate” at stalled replication forks, coordinating its nuclease and ATPase activities to overcome replication stress.

We show that DinG binds to lssDNA with a higher affinity than its exonuclease-deficient DinG D10 E12A mutant (Supplementary Figure S4), and even surpasses the ssDNA affinity reported for DinG_Sau_ [11]. The ssDNA-dependent ATPase activity of DinG is neither stimulated nor inhibited by concurrent lssDNA degradation, indicating that ATP binding or hydrolysis does not switch off its exonuclease function on lssDNA (Figure 2 and Figure 3). Furthermore, DinG can degrade the RNA strand of RNA–DNA hybrids and remove R-loops from genomic DNA, activities resembling those of RnhC and PcrA. Unlike DinG_Sau_ in the presence Mg^2+^ [10], DinG does not degrade lssRNA unless Mg^2+^ is substituted for Mn^2+^ (Figure 1C). Furthermore, on unreplicated fork DNA, apo DinG binds and degrades the 3′-tail, destabilising the junction and continuing exonucleolytic degradation of the 3′-ended strand. Conversely, DinG·ATP binds and degrades the 3′-tail but it may dissociate from fork DNA before reaching the duplex junction (Figure 5B), suggesting that ATP- or ADP-bound states do not inhibit its exonuclease activity.

In unstressed, exponentially growing cells, the replisome frequently encounters arrays of RNAPs transcribing highly expressed genes in the CD orientation as well as R-loops formed there. These obstacles cause transient fork pausing or even replisome disassembly [15,16,19], generating ssDNA regions coated by SsbA reviewed in [36], and references therein, [52]. RecO then interacts with and partially displaces SsbA, and in concert with RecR promotes RecA nucleation at stalled forks [53,54]. Live-cell imaging studies have shown that in response to CD RTCs, RecA forms spontaneous foci that colocalise with DnaX and with *rrn* loci [15,17,19]. Similarly, RhnC, PcrA, and DinG each interact with RecA and form foci that spontaneously colocalise with replisome markers and with *rrn* loci (Figure 9) [18,19,21]. It is likely that, at RTCs, RNAP or RecA—alone or in concert—recruit specialised nucleases (RnhC, DinG) and DNA helicases (PcrA, RecG) that contribute to overcome R-loops (Figure 8A,C) and remodel stalled forks via error-free DNA damage tolerance sub-pathways (reviewed in [36]). If these error-free DDT sub-pathways are saturated, and the replicative DNAP cannot circumvent an endogenous lesion, PolC is replaced by PolY1 and PolA, which bypass the lesion via TLS, allowing replicative DNA synthesis to resume.

In response to exogenous threats (e.g., upon UV-, 4NQO-, or MMS-treatment), local and general replication stress responses (e.g., SOS response) are induced, leading to the enrichment of RecA and DinG foci at or near replication forks (Figure 9) [17]. RecA interacts with and may load fork remodellers to process stalled forks via error-free DNA damage tolerance sub-pathways [36]. If these mechanisms fail, lesions are bypassed by TLS DNAPs. DinG, assembled at the stalled forks, alongside RecA, PolA, and DnaN, contributes to lesion bypass by poorly understood mechanisms (Supplementary Figure S10). PolA, alone or in concert with DnaN, recruits PolY1 [21,22] and/or PolY2, although the molecular basis of TLS polymerase choice remains unknown. It has been proposed that PolY1 and PolY2 catalyse nucleotide incorporation opposite damaged template bases—yet often erroneously—which are subsequently transiently extended by PolA before the replicative PolC resumes high-fidelity synthesis [36], and references therein.

Elucidating how DinG recognises its substrates (stalled forks, RTCs, or RNA–DNA hybrids) and how these interactions activate its 3′→5′ exoribonuclease activity remains an important challenge for future work.

## 4. Materials and Methods

### 4.1. Bacterial Strains and Plasmids

The *B. subtilis* wt BG214 strain and its isogenic derivatives are listed in Supplementary Table S1. To construct the ∆*ypvA* mutant, the regions upstream and downstream of the *ypvA* coding region were amplified and fused to the *ermC* gene to form the ∆*ypvA*::*ermC* cassette. The entire gene to be characterised was subsequently replaced by the cassette through natural chromosomal transformation, with selection for erythromycin resistance (Supplementary Table S1).

Codons 10 and 12 of *dinG*, encoding for Asp and Glu, respectively, or codon 290, encoding for Lys, were replaced with Ala codons by site-directed mutagenesis (Supplementary Figure S1B,C), rendering the *dinG* D10A E12A and *dinG* K290A mutant genes. The wt *dinG*, *dinG* D10A E12A, or *dinG* K290A gene was subsequently cloned into NdeI-linearised pET3a, rendering the pCB1057, pCB1259, and pCB1260 plasmids, respectively. The *rnhC* gene was cloned into NdeI-linearised pET21b, resulting in the pCB1242 plasmid, which added a His-tag at the C-terminus of the protein (Supplementary Table S1). *E. coli* BL21(DE3) [pLysS] cells carrying these plasmids, along with *E. coli* M15 (pREP4) cells harbouring pCB1229 (*pcrA*), were used to overproduce and purify the DinG, DinG D10A E12A, DinG K290A, RnhC, or PcrA proteins (Supplementary Table S1). Additionally, *B. subtilis* JH642 (chromosomal based-*rpoC* His-tagged) and BG214 carrying pBT61 (*recA*) were used to overproduce and purify the RNAP holoenzyme and RecA, respectively (Supplementary Table S1).

The wt *dinG*, *dinG* D10A E12A, *dinG* K290A, or *ypvA* gene was also cloned into the multicopy plasmid pDG148 [55], under the control of an isopropyl β-D-thiogalactopyranoside (IPTG)-inducible P*_spank_* promoter, for protein overexpression in *B. subtilis*. This process resulted in the pDinG, pDinG D10A E12A, pDinG K290A, and pYpvA plasmids, respectively (Supplementary Table S1).

To determine whether DinG colocalises with the replisome, mGold was fused to the 3′-terminus of the *dinG* gene, rendering the *dinG*-mGold gene fusion, in an otherwise wt background. Natural competent cells were then used to introduce the *dnaX*-CFP fusion on the *dinG*-mGold background.

The 3199 bp pGEM3 Zf(+)scDNA, the EcoRI- or KpnI-linearised pGEM3 Zf(+) ldsDNA, and the pGEM3 Zf(+) cssDNA were purified and used for in vitro assays, as earlier described [54]. As donor DNA for natural chromosomal transformation assays, the 2997 base pair (bp) coding region of the essential housekeeping *rpoB*482 gene, which encodes for the β subunit of RNAP, was selected. The *rpoB*482 gene carries a single C to T mutation at the centrally located codon 482 (at position 1443) that confers resistance to rifampicin (Rif^R^) [56]. The artificially oligomerised heterologous 4917 bp pHP14 plasmid DNA, which confers resistance to erythromycin (Em^R^), was used as donor DNA for natural plasmid transformation [56].

The *E. coli* BTH101 strain was used to analyse protein–protein interactions in vivo using bacterial adenylate cyclase two-hybrid assays (Supplementary Table S1). For this purpose, the T18 or T25 catalytic domain of the *Bordetella* adenylate cyclase gene, carried in the pUT18, pUT18C, pKT25, or pKNT25 vector, was fused to either the 5′ or 3′ termini of the *dinG*, *polA*, *polY1*, *polY2*, *dnaN*, or *recA* gene. The pKT25-zip and pUT18C-zip plasmids were used as positive controls, as described [57].

### 4.2. Cell Viability and Survival Studies

Cells carrying the empty vector (pDG148), pDinG, pDinG D10A E12A, pDinG K290A, or pYpvA plasmid were grown in LB medium at 37 °C with shaking, either with or without 125 μM IPTG. At OD_560_ = 0.4, cultures grown in the absence of IPTG were diluted and plated on LB agar with or without IPTG (125 μM), while cells grown in the presence of IPTG were diluted and plated on LB agar without IPTG. Plates were incubated overnight (16–18 h, 37 °C), and the number of colonies was counted. At least four independent experiments were performed, and colony-forming unit (CFU) data are presented as the mean ± standard error of the mean (SEM).

Cell survival following chronic exposure to DNA damage induced by MMS or H_2_O_2_ (Merck, Darmstadt, Germany) was assessed by growing cultures to OD_560_ = 0.4 at 37 °C with shaking, followed by plating appropriate dilutions on LB agar plates supplemented with the indicated concentrations of the DNA-damaging agent, as described [27]. Plates were incubated overnight (16–18 h, 37 °C), and the number of CFUs was counted. At least four independent experiments were conducted, and fractional survival data are presented as mean ± SEM.

### 4.3. Genetic Recombination Studies

The *B. subtilis* mutant strains listed in Supplementary Table S1 were brought to the natural competent state as previously described [58]. Competent cells (~5 × 10^8^ CFUs·mL^−1^) were incubated with either *rpoB*482 chromosomal DNA or pHP14 plasmid DNA (0.1 μg·mL^−1^) for 60 min at 37 °C. Then, the reaction mixture was plated on Rif (8 μg·mL^−1^) or Em (2 μg·mL^−1^) selective plates, respectively, and incubated (16–18 h, 37 °C), after which the number of transformants was counted [58]. An aliquot of competent cells was grown in parallel under identical conditions but without the addition of a plasmid, then diluted and plated on non-selective LB agar plates to determine the total cell count. At least four independent experiments were performed, and data are presented as the mean proportion of transformants relative to total cells ± SEM. Statistical significance was assessed using *t*-tests.

The size of the subpopulation that transiently develops natural competence can vary between 0.1% and 5% of the total cell population. To normalise DNA uptake between strains, the proportion of competent cells in each population was estimated by measuring the uptake of radiolabelled donor dsDNA, determined as the DNase I-resistant radiolabelled linear dsDNA in cells grown to competence, as described [58]. This normalisation process, however, introduces a degree of variability, so an increase or decrease in the number of transformants by <3-fold was considered poorly significant.

### 4.4. Mutagenesis Assays

Cultures were grown to OD_560_ = 0.8 at 37 °C with shaking in LB broth (30 cultures per condition). To determine the induced or spontaneous mutagenesis rate, cultures were either treated with 3 mM MMS (15 min) or left untreated, respectively, as described [48]. Cells (50 mL) were harvested by centrifugation, resuspended in LB, and plated onto LB agar plates containing Rif (8 μg·mL^−1^). The total number of viable cells was determined by plating on LB agar plates without antibiotic. Plates were incubated overnight (16–18 h, 37 °C), and CFUs were counted. Mutation frequencies were calculated as the number of Rif^R^ CFUs to the total number of CFUs. Statistical significance was assessed using *t*-tests.

### 4.5. In Vivo Protein–Protein Interaction Assays

The adenylate cyclase-based bacterial two-hybrid technique was employed as previously described [48,57]. Plasmid-borne fusions of DinG, PolA, PolY1, PolY2, DnaN, or RecA, to the N- or C-terminus of the T18 or T25 catalytic domain of *Bordetella pertussis* adenylate cyclase, were co-transformed with plasmid-borne fusions of PolA or DinG, to the N- or C-terminus of the T18 or T25 domain, into the reporter *E. coli* BTH101 strain. Empty vectors, as well as the pKT25-zip and pUT18C-zip vectors, were co-transformed into the reporter strain as negative and positive controls, respectively. Co-transformants were spotted onto LB plates supplemented with ampicillin, neomycin, streptomycin, 0.5 mM IPTG, and 10% X-gal. Plates were incubated for 3–4 days at 25° C. Each transformation was performed in at least triplicate, and a representative result is shown.

### 4.6. Single Particle Tracking and Diffusion Analysis

Cells were spotted on 25 mm coverslips (Epredia, Kalamazoo, MI, USA) and covered using 1% agarose pads. These pads were prepared with a fresh S750 minimal medium by sandwiching the agarose between two smaller 12 mm coverslips (Marienfeld, Lauda-Königshofen, Germany). Before use, all coverslips were cleaned by sonication in a Hellmanex II solution (1% *v*/*v*) for 15 min, followed by rinsing with distilled water and a second round of sonication in double-distilled water.

Slimfield microscopy was employed to visualise single fluorescent proteins at very high acquisition rates. This technique reduces the width of the laser beam to underfill the back aperture of the objective, creating a high-intensity light area approximately 10 μm in diameter. Single-molecule visualisation is achieved by photobleaching most of the fluorescent molecules in the cell (between 100 and 500 frames for medium-to-highly expressed proteins), followed by tracking the remaining and newly synthesised molecules for around 3000 frames. A 20 ms stream acquisition was performed using an Olympus IX-71 microscope, equipped with a high numerical aperture (NA) total internal reflection (TIRF) objective (UAPON × 100, Oil, NA = 1.49), a back-illuminated Electron-Multiplying Charge-Coupled Device (EMCCD) iXon Ultra camera (Andor Solis, Oxford Instruments, Oxon, UK) for rapid image capture, and a light-emitting diode (LED) laser LuxX 488-100 (488 nm, 100 mW) for fluorophore excitation. The laser beam was focused on the back focal plane and operated at up to 2 mW (60 W/cm^2^ at the image plane) during image acquisition. Andor Solis software (version 4.21) was used for imaging stream acquisition, with 3000 frames captured at 20 ms intervals.

Acquired image streams were uniformly cropped based on photobleaching curves, yielding 2000 frames, and adjusted for pixel sizes of 100 nm and time increments using Fiji [59]. Single-particle tracking was performed using u-track 2.2 [60], a software specifically developed for Matlab (MathWorks, Natick, MA, USA). Only trajectories with a minimum length of five steps were considered for further statistical analysis. Data analysis was conducted using a custom Matlab script, SMTracker 2.0, which is available upon request [61]. A Gaussian mixture model (GMM) was applied to fit the probability density distribution function of all frame-to-frame displacements, allowing for the determination of the standard deviations (σ_1_ and σ_2_), and the fractions (F_1_ and F_2_) of the static and mobile subpopulations of molecules, respectively.

A widely accepted method for analysing the diffusive behaviour of molecules involves plotting the mean squared displacement (MSD) versus time-lag curve. This approach provides estimates of both the diffusion coefficient and the kind of molecular motion (e.g., diffusive, sub-diffusive, or directed). However, this method assumes that each trajectory represents only one type of homogeneous motion and ideally requires trajectories of infinite lengths. Diffusion constants were calculated using the formula $Di=\;\frac{\sigma^{2}}{2\Delta t}\;(for\;i=1,2)$, where Δ*t* is the time interval between successive imaging frames. To differentiate between immobile and mobile molecules, frame-to-frame displacements in both the x and y directions were compared for all molecules [61]. Finally, trajectory maps and visualisations of static and mobile tracks in a standardised cell were generated using SMTracker 2.0, which is available upon request [61].

### 4.7. Enzymes, Reagents, and Protein Purifications

All chemicals used were of analytical grade (Merck, Darmstadt, Germany). RNase A, DNA polymerases, restriction enzymes, and DNA ligases were obtained from New England Biolabs (Ipswich, MS, USA), while protein purification matrices were supplied by Cytiva (Marlborough, MA, USA). The nucleotide sequences of the oligonucleotides used in this study (Merck, Darmstadt, Germany) are listed in Supplementary Table S2, and the assembled DNA structures are shown in Supplementary Figure S3. Concentrations of dsDNA, ssDNA, and ssRNA were determined using extinction coefficients of 0.020 μg·mL^−1^ cm^−1^, 0.027 μg·mL^−1^ cm^−1^, and 0.025 μg·mL^−1^ cm^−1^ at 260 nm, respectively. Concentrations are expressed either as moles of DNA molecules or moles of nucleotides, as indicated in the text.

*E. coli* BL21(DE3) [pLysS] cells harbouring pCB1057 (*dinG*), pCB1259 (*dinG* D10A E12A), or pCB1260 (*dinG* K290A) were grown for 2h at 30 °C after the addition of IPTG (1 mM) to induce protein expression. Cells were harvested by centrifugation, resuspended in buffer A (50 mM Tris HCl pH 7.5, 15% glycerol, 1 mM EDTA, 1 mM DTT) containing 600 mM NaCl, and lysed using a French Press. Following centrifugation, DinG was present in the soluble fraction. DNA and cell debris were removed by adding polyethyleneimine to a final concentration of 0.25% (A_260_ = 120), followed by centrifugation at 17,000× *g* for 45 min. The DinG-containing supernatant was precipitated with 70% (*w*/*v*) ammonium sulphate. The resulting pellet was resuspended in buffer A containing 100 mM NaCl and loaded onto a Q-Sepharose column equilibrated with the same buffer. The column was washed, and DinG was eluted using a linear gradient from 100 to 400 mM NaCl. Fractions containing DinG were pooled and subsequently loaded onto a Blue-Sepharose column. After column washing, DinG was eluted with buffer A containing a linear gradient from 200 to 1000 mM NaCl. The peak fractions containing DinG were pooled, concentrated using a hydroxyapatite column, and eluted with buffer A containing 100 mM KH_2_PO_4_/K_2_HPO_4_. DinG was dialysed at 4 °C and stored in buffer A containing 300 mM NaCl and 50% glycerol at −20 °C. The DinG D10A E12A and DinG K290A mutants were purified following the same protocol as the wt protein.

RnhC was purified from *E. coli* BL21(DE3) [pLysS] cells bearing pCB1242 (*rnhC*-His). Cells were cultured in LB medium at 30 °C to A_600_= 0.5, and protein expression was induced by adding 1 mM IPTG and incubating for an additional 3 h at 30 °C. Cells were harvested by centrifugation at 6000× *g* for 10 min at 4 °C, and then resuspended in buffer B (50 mM Tris-HCl pH 7.5, 1 M NaCl, 15% glycerol) containing 0.5% Brij-58. Cells were disrupted using a French Press, and cell debris was separated from the soluble lysate by centrifugation at 18,000× *g* for 45 min at 4 °C. The soluble RnhC extract was loaded onto a Ni^2+^-activated His-Trap chelating column that had been pre-equilibrated with 10 column volumes of buffer B containing 5 mM imidazole. RnhC was eluted using a linear gradient of buffer B containing from 20 to 100 mM imidazole. Fractions containing RnhC were analysed by SDS-polyacrylamide gel electrophoresis (PAGE). Purified RnhC was dialysed in buffer C (50 mM Tris-HCl pH 7.5, 300 mM NaCl, 1 mM DTT) containing 50% glycerol at 4 °C and stored at −20 °C. PcrA, RecA, and RNAP were purified as described [27,62,63], and were snap frozen before storage at −20 °C.

The purified proteins exhibited ~99% purity as assessed by SDS-PAGE. Full-length and partially proteolysed DinG variants were analysed by MALDI-TOF mass spectrometry to confirm the integrity and identity of the proteins, as well as to detect any potential contaminants. The molar extinction coefficients at 280 nm for DinG (and its mutant variants), RnhC, PcrA, RecA, and the RNAP holoenzyme were calculated as 73,020, 20,525, 70,375, 15,200, and 236,000 M^−1^ cm^−1^, respectively, as described [63]. Protein concentrations were determined based on these molar extinction coefficients, and the concentrations of DinG, its mutant variants, RnhC, PcrA, RecA, and the RNAP holoenzyme are expressed as moles of monomers.

### 4.8. ATPase Assays

The ATPase activity of DinG, DinG D10A E12A, and DinG K290A was measured via an ATP/NADH-coupled spectrophotometric enzymatic assay, by monitoring the decrease in absorbance at 340 nm, indicative of NADH conversion to NAD, using a Shimadzu CPS-20A (Kyoto, Japan) dual-beam spectrophotometer, as described [63].

Assays were conducted in buffer D (20 mM Tris-HCl pH 7.5, 50 mM NaCl, 3 mM MgCl_2_, 50 μg·mL^−1^ BSA, 4 mM DTT, 5% glycerol) containing 5 mM ATP and an ATP regeneration system (620 μM NADH, 100 U·mL^−1^ lactate dehydrogenase, 500 U·mL^−1^ pyruvate kinase, and 2.5 mM phosphoenolpyruvate) for 30 min at 37 °C. The concentration and order of addition of the purified proteins are detailed in the text. DNA effectors—including a 3199 nt pGEM3 Zf(+) scDNA, ldsDNA, cssDNA, or heat denatured pGEM3 Zf(+) ldsDNA to yield pGEM3 Zf(+) lssDNA—were added to the reaction mixture when indicated. Data obtained from A_340_ absorbance measurements were converted to ADP produced and plotted as a function of time. In some reactions, the curves reached a plateau due to the consumption of the NADH pool, and the rate of ATP hydrolysis was derived from the slope of the linear portion of the curves, as described [63]. At least three independent experiments were performed, and representative graphs with k_cat_ means ± SEM are shown. Statistical significance was analysed using *t*-tests.

### 4.9. Streptavidin Displacement, DNA Degradation, and DNA Unwinding Assays

For streptavidin (SA) displacement assays, a synthetic linear 45-mer oligodeoxynucleotide (lssDNA_45_), linked to a single biotin at the second inter-nucleotide from the 3′-end was 5′-end-labelled with γ^32^P-ATP using T4 polynucleotide kinase and purified, yielding a 3′-Biotin γ^32^P-lssDNA_45_ (Supplementary Table S2). When indicated, the 3′-Biotin γ^32^P-lssDNA_45_ (0.5 nM in DNA molecules) was pre-incubated with saturating streptavidin (2.5 nM) in buffer D for 5 min at 37 °C to form SA-3′-Biotin γ^32^P-lssDNA_45_ complexes. The free 3′-Biotin γ^32^P-lssDNA_45_ or SA-3′-Biotin γ^32^P-lssDNA_45_ was then incubated with increasing concentrations of DinG or its mutant variants (DinG K290A or DinG D10A E12A) in buffer D, either containing or lacking 2.5 mM ATP, for 5 min at 37 °C. The reaction products were analysed by native 10% PAGE in 90 mM Tris-borate 2.5 mM EDTA (TBE 1X) and visualised by autoradiography. At least three independent experiments were performed, and representative gels are shown.

For DNA degradation and unwinding assays, the plasmid-size scDNA, ldsDNA, and cssDNA were prepared as described earlier [54], and the synthetic DNA and RNA oligonucleotides (Supplementary Table S2) were 5′-end-labelled, purified, and used to assemble various DNA substrates (Supplementary Figure S3), as described previously. Of note, short DNA or RNA molecules exhibit anomalous migration patterns when analysed under denaturing PAGE conditions. The DNA substrates were incubated with a fixed amount of DinG (or its variants) for a variable time, or with increasing concentrations of DinG for a fixed time, in buffer D, with or without 2.5 mM ATP. When indicated, 3 mM MgCl_2_ was replaced by 3 mM MnCl_2_ or 3 mM CaCl_2_. Reactions were deproteinised, and the reaction products were analysed using 0.8% agarose gels, or under native (15% PAGE in TBE 1X) or denaturing (20 or 16% PAGE in TBE 1X containing 8 M urea) conditions. Gels were imaged using a GelDoc Imaging System (Bio-Rad, Hercules, CA, USA), or autoradiographed. At least three independent experiments were performed, and representative gels are presented.

### 4.10. Protein–DNA Interactions

DinG binding to DNA substrates was assessed using EMSAs. Briefly, the 3′-Biotin γ^32^P-lssDNA_45_ substrate (0.5 nM, in DNA molecules) was incubated with increasing concentrations of DinG or its mutant variants for 10 min at 37 °C in buffer D. When indicated, 3 mM MgCl_2_ was replaced by 2 mM EDTA. The reaction products were analysed under native conditions (15% PAGE in TBE 1X). Gels were autoradiographed, and the apparent binding constant (K_app_) values were calculated. At least three independent experiments were performed, with representative gels presented.

### 4.11. R-Loop Removal Analyses

BG1751 (∆*rnhC*) cells were grown in LB medium with agitation to an OD_560_ = 0.4. Following this, 20 mM MMS was added for 15 min, after which the culture was harvested by centrifugation at 4 °C. The cells were treated with lysozyme (1 mg·mL^−1^), and the lysate was treated with RNase A (0.1 mg·mL^−1^) and proteinase K (1 mg·mL^−1^) for 60 min at 37 °C to remove RNA and proteins. gDNA was purified using phenol–chloroform–isoamyl alcohol extraction (25:24:1) followed by ethanol precipitation. The purified gDNA (650 μM in nt) was incubated with increasing concentrations of the indicated protein for 15 min at 37 °C in buffer E (10 mM Tris-HCl [pH 7.4], 50 mM NaCl, 10 mM MgCl_2_, 1 mM DTT). When indicated, 5 mM ATP was added to buffer E. After incubation, samples were serially diluted in PBS and 2 µL were spotted onto a positively charged Hybond-N^+^ nylon membrane (Cytiva, Marlborough, MA, USA). The membranes were air-dried for 30 min, then UV-cross-linked at 0.12 J·m^−2^, and blocked in a TBST buffer (50 mM Tris-HCl [pH7.4], 100 mM NaCl, 0.1% [*v*/*v*] Triton) containing 5% milk for 120 min at 22 °C. Detection of R-loops was performed by probing the membranes with S9.6 mouse mAb (1:1000 dilution, MABE1095, Millipore, Darmstadt, Germany) in TBST containing 1% BSA overnight at 4 °C. An anti-mouse HRP (Santa Cruz Biotechnology, Dallas, TX, USA) was used as the secondary antibody (1 h, 22 °C, 1:5000 dilution). Signal acquisition was performed using a ChemiDoc Touch Imaging System (Bio-Rad, Hercules, CA, USA), and dot signals were quantified using Image Lab 3.0 software (Bio-Rad, Hercules, CA, USA). At least three independent experiments were performed, and representative membranes are presented.

### 4.12. In Vitro Protein–Protein Interaction Assays

Protein–protein interactions were analysed using immuno-dot blot assays [64]. Briefly, increasing amounts of RecA (when indicated), DinG, RnhC, PcrA, RNAP, and Bovine serum albumin (BSA) as a negative control were applied to a pre-wetted nitrocellulose membrane in phosphate-buffered saline (PBS) buffer (137 mM NaCl, 2.7 mM KCl, 8 mM Na_2_HPO_4_, and 2 mM K_2_PO_4_ [pH 7.4]). Following a blocking step with PBS containing 5% (*w*/*v*) skimmed milk powder, the membrane was incubated for 6 h at 4 °C with 200 ng·ml^−1^ RecA in a binding solution (PBS, 0.5% [*w*/*v*] skimmed milk powder and 0.1% [*v*/*v*] Triton X-100), or not—for antibody specificity assessing. The membrane was extensively washed and incubated overnight at 4 °C with an anti-RecA polyclonal antibody (dilution 1:6000), followed by a 60 min incubation at 22 °C with a peroxidase-conjugated anti-rabbit IgG secondary antibody (Cytiva, Marlborough, MA, USA, Ref: NXA931, dilution 1:5000). The interactions were visualised using the Clarity^TM^ Western ECL Substrate kit (Bio-Rad, Hercules, CA, USA). Images were captured and processed with the ChemiDoc Touch Imaging System and the Image Lab 3.0 software (Bio-Rad, Hercules, CA, USA). Each interaction was tested at least three times, and representative membranes are presented.

## Figures and Tables

**Table 1:** Diffusion constants of static and mobile fractions of DinG-mGold molecules.

Condition	N^o^ Cells	N^o^ Tracks	D ^a^	D_1_ ^b^	D_2_ ^c^
-	138	25,841	0.2160 ± 0.018	0.043 ± 0.002	0.67 ± 0.002
+ MMS	131	14,608	0.1770 ± 0.011	0.043 ± 0.001	0.67 ± 0.002
+ Rif	133	12,031	0.0980 ± 0.017	0.043 ± 0.001	0.67 ± 0.001

^a^ D, MSD, average diffusion constant of all molecules (µm^2^·s^−1^). ^b^ D_1_, diffusion constant of static fraction (µm^2^·s^−1^). ^c^ D_2_, diffusion constant of mobile fraction (µm^2^·s^−1^).

## Data Availability

All data necessary to evaluate the conclusions presented in this paper are available within the main text and/or Supplementary Materials. Raw gel images, ATPase data, and materials utilised in this study are maintained by the authors and can be provided upon request.

## Supplementary Materials

The supporting information can be downloaded at: https://www.mdpi.com/article/10.3390/ijms26199681/s1. References [65,66,67,68] are cited in the Supplementary Materials.
