# Supplementary material for: Bacillus subtilis DinG 3′⟶5′ Exo(ribo)nuclease: A Helpmate to Mitigate Replication Stress
Source: Int J Mol Sci. 2025 Oct 4;26(19):9681. doi: 10.3390/ijms26199681 (PMC12525004; doi:10.3390/ijms26199681)
Supplement: Supplementary file 1 [file ijms-26-09681-s001.zip › ijms-3898601-supplementary.pdf]

Supplementary Materials

Bacillus subtilis DinG 3′ → 5′ exo(ribo)nuclease: A helpmate to mitigate replication stress

Begoña Carrasco[1], Rubén Torres[1], María López-Sanz[1]; Rogelio Hernández-Tamayo[2,3], Peter L. Graumann[2,4], Juan C. Alonso[1]

1 Department of Microbial Biotechnology, Centro Nacional de Biotecnología (CNB-CSIC), Calle Darwin 3, 28049, Madrid, Spain; bcarrasc@cnb.csic.es (B.C.), rtorres@cnb.csic.es (R.T.), mlopez@cnb.csic.es (M.L.-S.), jcalonso@cnb.csic.es (J.C.A.)

2 Center for Synthetic Microbiology (SYNMIKRO), Karl-von-Frisch-Straße 14, 35043, Marburg, Germany; rogelio.hernandez@synmikro.uni-marburg.de (R.H.-T.); graumanp@uni-marburg.de (P.L.G.)

3 Max Planck Institute for Terrestrial Microbiology, Karl-von-Frisch-Straße 10, 35043, Marburg, Germany

4 Department of Chemistry, University of Marburg, Hans-Meerwein-Straße 4, 35032, Marburg, Germany

This supplementary file contains:

Sections (A and B).

Tables (S1 and S2).

Figures (S1 to S10).

Section A. *dinG inactivation does not significantly impair homologous recombination*

Many of the *E. coli* ssDNA-specific 3′→5′ exonucleases, which are crucial for removing dirty ends during basal end-resection [1], are absent in *B. subtilis*. However, DinG possesses a DEDD exonuclease domain that shares significant sequence identity with XonA$_{Eco}$, a 3′→5′ exonuclease involved in basal end resection (Supplementary Figure S1A). To determine whether DinG contributes to either basal end resection or long-range end resection during DSB repair, *via* AddAB, RecJ-RecQ or RecJ-RecS complexes, the Δ*dinG* mutation was moved into Δ*addAB*, Δ*recJ*, Δ*recQ*, or Δ*recS* strains, strains specifically deficient in end resection (Supplementary Table S1) [2]. Subsequently, we examined the effects of DinG loss on genetic recombination (natural transformation) and repair-by-recombination.

First, we measured natural chromosomal transformation, which occurs independently of DNA replication and transcription. In this process, the DNA uptake apparatus of a natural competent cell translocates linear ssDNA into the cytosol, where it is immediately coated by the single-stranded binding proteins SsbA and SsbB. Then, RecA, aided by the DprA mediator (or RecO in Δ*dprA* cells), filaments on it [3, 4]. Once a minimal efficient processing segment is identified in the haploid resident genome, the RecA nucleoprotein filament invades the homologous duplex DNA, leading to the formation of a three-strand paranemic displacement loop (D-loop). RecA then catalyses homology-directed DNA strand-exchange with the assistance of the RadA/Sms DNA helicase [5]. Finally, the displaced strand of the D-loop intermediate is nicked and subsequently degraded by end-resection functions, enabling ligation of the incoming lssDNA to the resected recipient strand, thus leading to the integration of the transforming DNA in the chromosomal duplex [4]. Plasmid transformation, in which heterologous plasmid ssDNA is established as an episome in the recipient cell *via* a process independent of end-resection and RecA [4], was evaluated as a control.

Cells bearing the Δ*addAB*, Δ*recS*, Δ*recQ* or Δ*recJ* mutation, either alone or in concert with the Δ*dinG* mutation, were made naturally competent to analyse genetic recombination. Chromosomal transformation was not impaired in Δ*dinG* competent cells and also remained unaffected in Δ*recS* and Δ*addAB* cells ($p > 0.1$) (Supplementary Figure S7A), as previously observed [6]. Chromosomal transformation was significantly impaired in Δ*recJ* and, to a lesser extent, in Δ*recQ* cells ($p < 0.01$) (Supplementary Figure S7A), as previously described [6]. The double mutants exhibited the same phenotype as their most affected relative parental strain ($p > 0.1$) (Supplementary Figure S7A). It is likely that the RecJ 5′→3′ ssDNA exonuclease, in concert with the RecQ DNA helicase, degrades the displaced strand at the D-loop recombination intermediate, as earlier proposed [6]. Heterologous plasmid transformation was only marginally impaired in Δ*dinG* Δ*recS* competent cells (Supplementary Figure S7A).

Secondly, we examined whether DinG is required for DSB and single-strand gap repair. To do so, the single and double mutant strains were grown in LB medium to an OD_{560} = 0.4 at 37 ºC with shaking. Appropriate dilutions were then plated on LB agar plates containing MMS or H_2O_2. Both agents induce template base lesions that are specifically removed by base excision repair, but if left unrepaired can cause replicative stress. H_2O_2 can also induce nicks that may

lead to replication fork collapse, resulting in double-strand breaks [7, 8]. As shown in Supplementary Figure S7BC, Δ*dinG* cells remained proficient in recombination and apparently as capable of repairing alkylating or oxidative DNA damage as wt cells. Δ*recQ* cells also remained unaffected. In contrast, Δ*recJ* and Δ*recS* cells were sensitive to alkylating DNA damage, and Δ*addAB* cells were very sensitive to both alkylating and oxidative DNA damage [2]. The Δ*dinG* Δ*recQ*, Δ*dinG* Δ*recS*, Δ*dinG* Δ*recJ* or Δ*dinG* Δ*addAB* double mutants were as sensitive as their most sensitive single parental mutant (Supplementary Figure S7B–C).

Therefore, the absence of DinG does not alter the phenotypes of mutants deficient in functions related to end-resection. Collectively, these data suggest that DinG neither contributes to basal- or long-range end resection, nor to degrade the displaced strand at D-loops during natural chromosomal transformation. However, we cannot rule out the possibility that functional redundancies with the YpvA paralogue may hinder the role of DinG (see Introduction).

Section B. *DinG or YpvA overexpression differentially affects cell proliferation*

To further investigate the role of DinG *in vivo*, we assessed whether the overexpression of wt *dinG* or *ypvA* affects cell proliferation. To evaluate the significance of DinG domains, we also analysed overexpression of the two mutant variants: DinG D10A E12A (exonuclease-deficient) and DinG K290A (ATPase-deficient). Each gene was cloned under the IPTG-inducible promoter P_{spank} on the Nm^R multicopy pDG148 plasmid, resulting in pDinG, pDinG D10A E12A, pDinG K290A, and pYpvA (Supplementary Table S1). The empty pDG148 plasmid was included as a control.

BG214 wt cells bearing pDinG, pDinG D10A E12A, pDinG K290A, pYpvA, or the empty vector were grown at 37 ºC in LB medium supplemented with Nm (10 $\mu g \cdot ml^{-1}$), either with or without 125 μM IPTG. In the absence of IPTG, all strains exhibited similar doubling time (33-36 min), suggesting that any potential leaky expression from the IPTG-inducible promoter did not significantly affect growth (Supplementary Figure S9A–B). In the presence of IPTG, which induces a significant overexpression of the DinG and YpvA proteins (Supplementary Figure S9C), cells bearing pDinG, pDinG K290A or pYpvA grew slower with a significant increase in doubling time. In contrast, cells carrying the empty vector or pDinG D10A E12A grew similarly to those in the absence of IPTG (Supplementary Figure S9A–B). These results suggest that: i) overexpression of DinG or YpvA compromises cell growth; and ii) the growth defect resulting from DinG overexpression is attributed to the DEDD exonuclease domains rather than to its ATPase domain.

To assess whether the overexpression of DinG or YpvA affects plating efficiency, cells at an OD_{560} = 0.4 were serially diluted and plated on LB agar plates without IPTG. Overexpression of DinG or YpvA in the presence of IPTG resulted in a significant reduction in the number of CFUs (Supplementary Figure S9D). In cells overexpressing DinG, we observed tiny and small size colonies, comprising 30% to 40% of the total CFUs (Supplementary Figure S9E). However, we were unable to grow these tiny and small colonies in LB medium containing Nm and IPTG.

Therefore, we wondered whether DinG overexpression induces a persistence state. To evaluate this hypothesis, cells were grown to an OD_{560} = 0.4 in the absence of IPTG, then serially diluted and plated on LB agar with or without IPTG. Here, overexpression of DinG or YpvA compromised CFU formation when compared both to the empty vector control or to the uninduced cultures, although this effect was less pronounced than when cultures were grown in LB medium with Nm and IPTG (Supplementary Figure S9D–F). This suggests that if persistence is induced, it is a minor phenomenon.

Table S1. Strains.

Strains	Relevant genotype	Source
BG214[a]	parental strain	Laboratory strain
BG425	+ Δ*recS*	[2]
BG675	+ Δ*recJ*	[2]
BG705	+ Δ*recQ*	[2]
BG1337	+ Δ*addAB*	[9]
BG1873	+ Δ*recA*	[10]
BG1749	+ Δ*rnhB*	[9]
BG1751	+ Δ*rnhC*	[9]
BG1605	+ Δ*dinG*	[11]
BG2049	+ Δ*ypvA* Δ*dinG*	This work
RH3700	+ *dinG*-mGold	This work
RH3701	+ *dinG*-mGold *dnaX*-CFP	This work
BG1973	+ Δ*ypvA*::Em^R	This work
BG1671	+ Δ*dinG* Δ*recA*	This work
BG1915	+ Δ*dinG* Δ*recJ*	This work
BG1673	+ Δ*dinG* Δ*recS*	This work
BG1675	+ Δ*dinG* Δ*recQ*	This work
BG1677	+ Δ*dinG* Δ*addAB*	This work
BG1905	+ Δ*dinG* Δ*rnhB*	This work
BG1907	+ Δ*dinG* Δ*rnhC*	[9]
JH642[b]	+ *rpoC*-His tagged	[12]

Strains	Relevant plasmids	Source
BG214	+ pDG148, P_{spac}-*lacI*, Nm^R, Ap^R (vector)	[13]
BG214	+ pDinG (*dinG*), Nm^R	This work
BG214	+ pDinG D10A E12A (*dinG* D10A E12A), Nm^R	This work
BG214	+ pDinG K290A (*dinG* K290A), Nm^R	This work
BG214	+ pYpvA (*ypvA*), Nm^R	This work
BG1973	+ pHP14, Cm^R, Em^R	This work
BG1973	+ pCB1310-Δ*dinG*::Nm^R, Cm^R, Em^R	This work
BG214[c]	+ pBT61 (*recA*), Nm^R	[14]
BL21(DE3)[d]	+ pCB1057 (*dinG*), pLysS, Ap^R	This work
BL21(DE3)[d]	+ pCB1259 (*dinG* D10A E12A), pLysS, Ap^R	This work
BL21(DE3)[d]	+ pCB1260 (*dinG* K290A), pLysS, Ap^R	This work
BL21(DE3)[d]	+ pCB1242 (*rnhC*), Ap^R	This work
M15[d]	+ pCB1229 (*pcrA*), pREP4, Ap^R	[9]
BTH101[e]	+ pUT18, pUT18C, pKT25, or pKNT25	[15]

[a]The genotype of the *B. subtilis* BG214 strain is: *trpCE metA*5 *amyE1 ytsJ*1 *rsbV*37 *xre*1 *xkd*A1 *att*$^{SP\beta}$ *att*^{ICEBs1}. [b]Only the relevant genotype of the *B. subtilis* strain used to purify RNAP is shown. [c]Strain and plasmid used to overexpress and purify RecA. [d]*E. coli* strains and plasmids used to overexpress and purify DinG, DinG D10A E12A, DinG K290A, RnhC and PcrA, respectively. [e]*E. coli* BTH101 cells bearing pUT18, pUT18C, pKT25, or pKNT25 containing the full-length genes of interest were used for bacterial adenylate cyclase two-hybrid assays. Abbreviations: Ap^R, ampicillin resistance; Cm^R, chloramphenicol resistance, Em^R, erythromycin resistance, and Nm^R, neomycin resistance.

Table S2. Sequence of the oligonucleotides.

Oligo	Nucleotide sequence
3´-Biotin lssDNA_{45}	5´-GTACGTATTCAAGATACCTCGTACTCTGTACTGACTCGGATCC(bioT)A-3´
ssDNA_{38} 201	5´-GCTCTGATGCCGCATAGTTAAGCCAGCCCCGACACCCG-3´
ssDNA_{38} 202	5´-CGGGTGTCGGGGCTGGCTTAACTATCGGCGATCAGAGC-3´
5´-overhang 203	5´-CGGGTGTCGGGGCTGGCTTAACTATG-3´
3´-overhang 204	5´-CTGGCTTAACTATGCGGCATCAGAGC-3´
ssRNA_{38} 206	5´-GCUCUGAUGCCGCAUAGUUAAGCCAGCCCCGACACCCG-3´
J170	5´-AGACGCTGCCGAATTCTGGCTTGGATCTGATGCTGTCTAGAGGCCTCCAC-TATGAAATCG-3´
J171	5´-CGATTTCATAGTGGAGGCCTCTAGACAGCA-3´
J172	5´-TGCTGTCTAGAGACTATCGATCTATGAGCT-3´
J173	5´-AGCTCATAGATCGATAGTCTCTAGACAGCATCAGATCCAAGCCAGAATTCGG-CAGCGTCT-3´

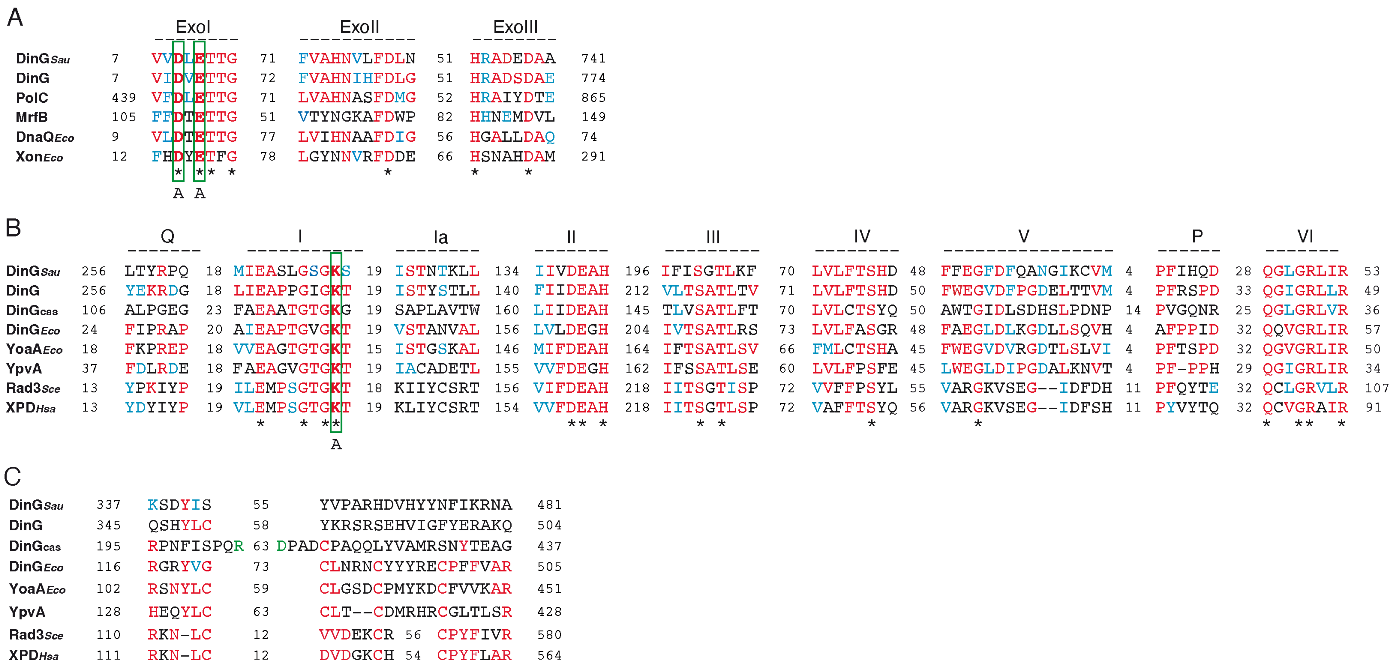

Figure S1. Amino acid sequence alignment of domains from representative proteins of different bacterial species: exonuclease domains (Exo I to III) (A), relevant helicase domains (B), and FeS cluster-binding motif (C). The protein origin is described in the text. The lengths separating the subdomains and distance to the N- or C-terminus are indicated. Relevant residues involved in the formation of a salt bridge within the FeS variant (vFeS) are highlighted in pale green. Identical residues are highlighted in red, conserved substitutions in blue, and highly conserved residues are marked by asterisks below the sequences. Mutated residues, substituted with Ala in the ExoI domain or Walker A box, are bolded and framed.

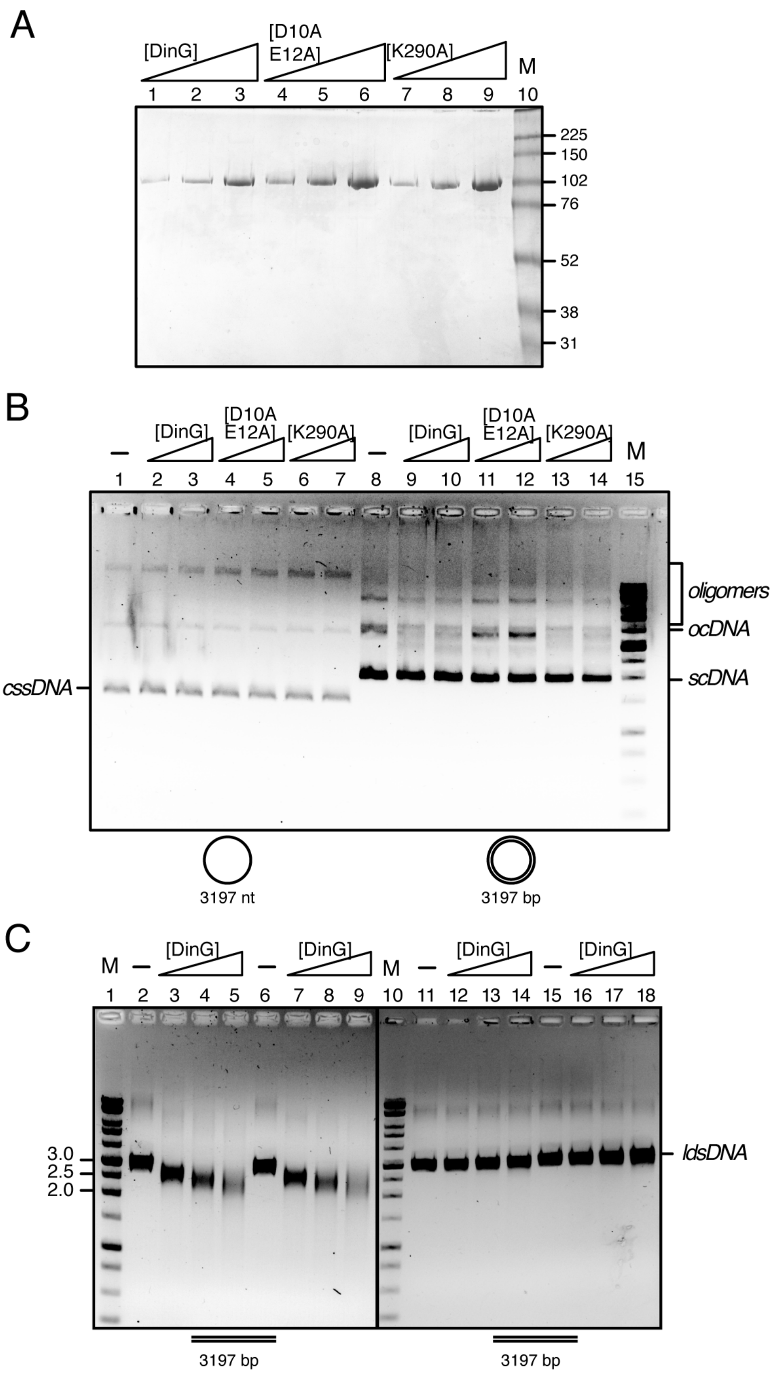

Figure S2. Purification and nuclease activity of DinG. (**A**) Increasing concentrations of purified wt DinG, Exo I mutant DinG D10A E12A, and ATPase motif I mutant DinG K290A were analysed by SDS-PAGE (10%). The molecular weight of the protein markers is indicated in kDa. (**B**) DinG, DinG D10A E12A, or DinG K290A (175 and 350 nM) was incubated with 3.1 nM (in DNA molecules) 3,199 nt pGEM3 Zf(+) cssDNA, or with supercoiled pGEM3 Zf(+) dsDNA for 15 min at 37 ºC in buffer D. (**C**) DinG (87.5─350 nM [lanes 3─5 and 7─9] or 0.87─3.5 nM [lanes 11─13 and 15─17]) was incubated with 3.5 nM (in DNA molecules) KpnI- (lanes 3-5 and 12-14) or EcoRI-linearised pGEM3 Zf(+) dsDNA (lanes 7-9 and 16-18) for 15 min at 37 °C in buffer D. Products were separated by 0.8% agarose gel electrophoresis. The length of the relevant DNA fragments is indicated in nt. "–", no protein added; "M", molecular weight marker; solid lines, DNA; and number, length of each strand (in nt). The position of the substrates is indicated. All reactions were repeated at least three times with consistent results, and representative gel images are shown.

Name	Substrate description	Structure + oligonucleotide composition
3´-biotin ssDNA	45-nt lssDNA with biotin at 3´-end	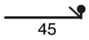
ssDNA (201)	38-nt lssDNA	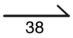
ssDNA (202)	38-nt lssDNA	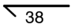
ssDNA (203)	26-nt lssDNA	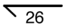
ssDNA (204)	26-nt lssDNA	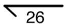
ssRNA(206)	38-nt lssRNA	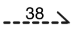
hybrid RNA-DNA (206+202)	38-nt RNA-DNA hybrid	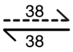
5´-tailed dsDNA (201+203)	5’-overhang	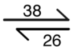
3´-tailed dsDNA (201+204)	3’-overhang	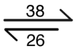
5´-tailed RNA-DNA (206+203)	5’-overhang RNA-DNA hybrid	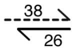
3´-tailed RNA-DNA (206+204)	3’-overhang RNA-DNA hybrid	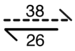
unreplicated fork (J170+J173)	30-nt dsDNA 30-nt ssDNA	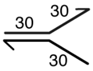
replicated fork (J170+J173+J171)	60-nt dsDNA 30-nt ssDNA	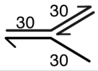
replicated fork (J170+J173+J172)	60-nt dsDNA 30-nt ssDNA	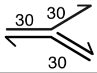

Figure S3. DNA substrates used in the assays. Substrates were constructed by annealing the indicated oligonucleotides (see Table S2). Solid lines represent DNA molecules, while dashed lines indicate RNA molecules. Arrowheads indicate the 3´-end, and a dot marks the biotin located at position dT_{44}. Numbers indicate the length of each strand (in nt).

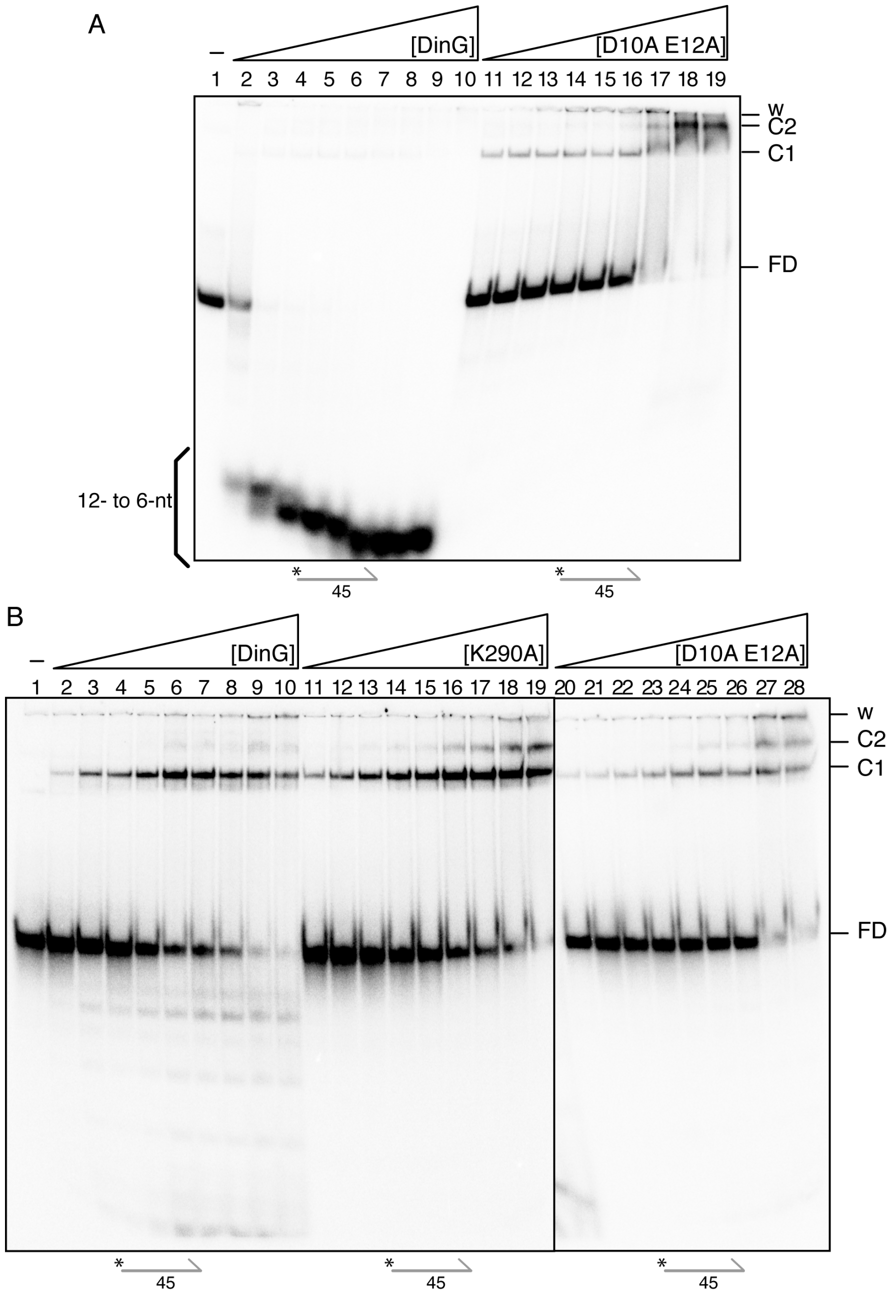

Figure S4. DinG binding and degradation of lssDNA under Mg^{2+} versus EDTA conditions. (**A**-**B**) 3′-Biotin $\gamma^{32}P$-lssDNA$_{45}$ (0.5 nM in DNA molecules) was incubated with increasing concentrations of wt DinG, DinG K290A, or DinG D10A E12A (0.08–20 nM) for 10 min at 37 ºC in buffer D (**A**) or in buffer D supplemented with 2 mM EDTA (**B**). The reaction mixture was then separated by native PAGE. "– ", no protein added; "FD", free lssDNA; "C1", first protein–lssDNA complex; "C2", second protein–lssDNA complex; "w", well; grey line, 5′-end labelled strand; solid line, DNA; "*", labelled nucleotide; arrowhead, 3′-end; and number, length of each strand (in nt). All reactions were repeated at least three times with consistent results, and representative gel images are shown.

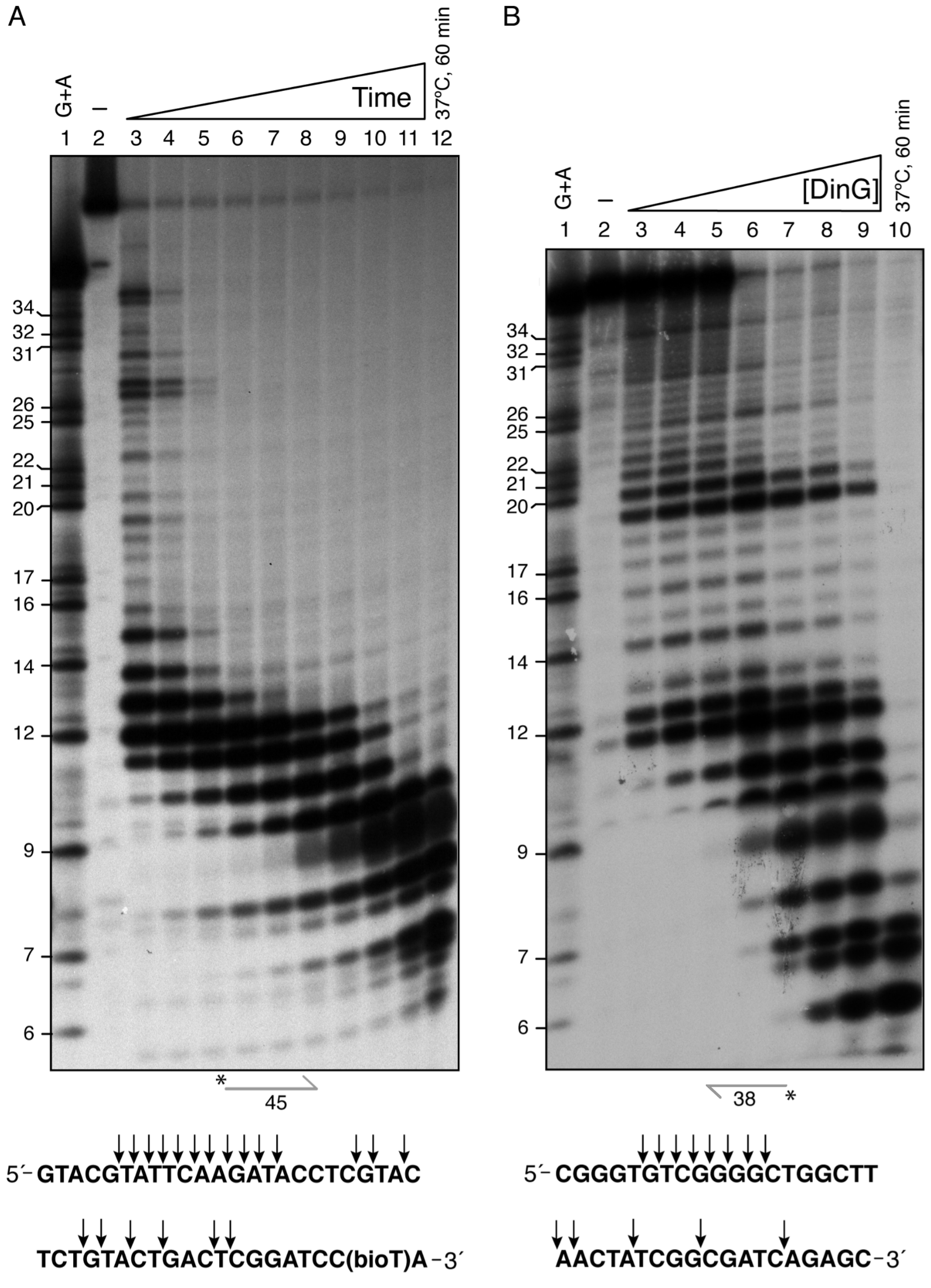

Figure S5. DinG pauses on lssDNA. (**A**) γ^{32}P-lssDNA$_{45}$ (5 nM in DNA molecules) was incubated with DinG (1 nM) for a variable time (0.5, 1, 2, 3.5, 5, 10, 15, 30 and 60 min) at 22 ºC (lanes 3—11) or for 60 min at 37 ºC (lane 12) in buffer D. (**B**) γ^{32}P-lssDNA$_{38c}$ (5 nM in DNA molecules) was incubated with increasing concentrations of DinG (1—60 nM) for 10 min at 22 ºC (lanes 3—9), or with 1 nM DinG for 10 min at 37 ºC (lane 10) in buffer D. The reaction mixtures were then analysed by denaturing gel electrophoresis. "–", no protein added; "G + A", mobility marker, with the molecule lengths indicated in nt (lane 1); grey line, 5′-end labelled strand; solid line, DNA; "*", labelled nucleotide; arrowhead, 3′-end; and number, length of each strand (in nt). Arrows indicate cleavage sites on the lssDNA sequence, inferred from the accumulation of intermediates and products. All reactions were repeated at least three times with consistent results, and a representative gel image is shown.

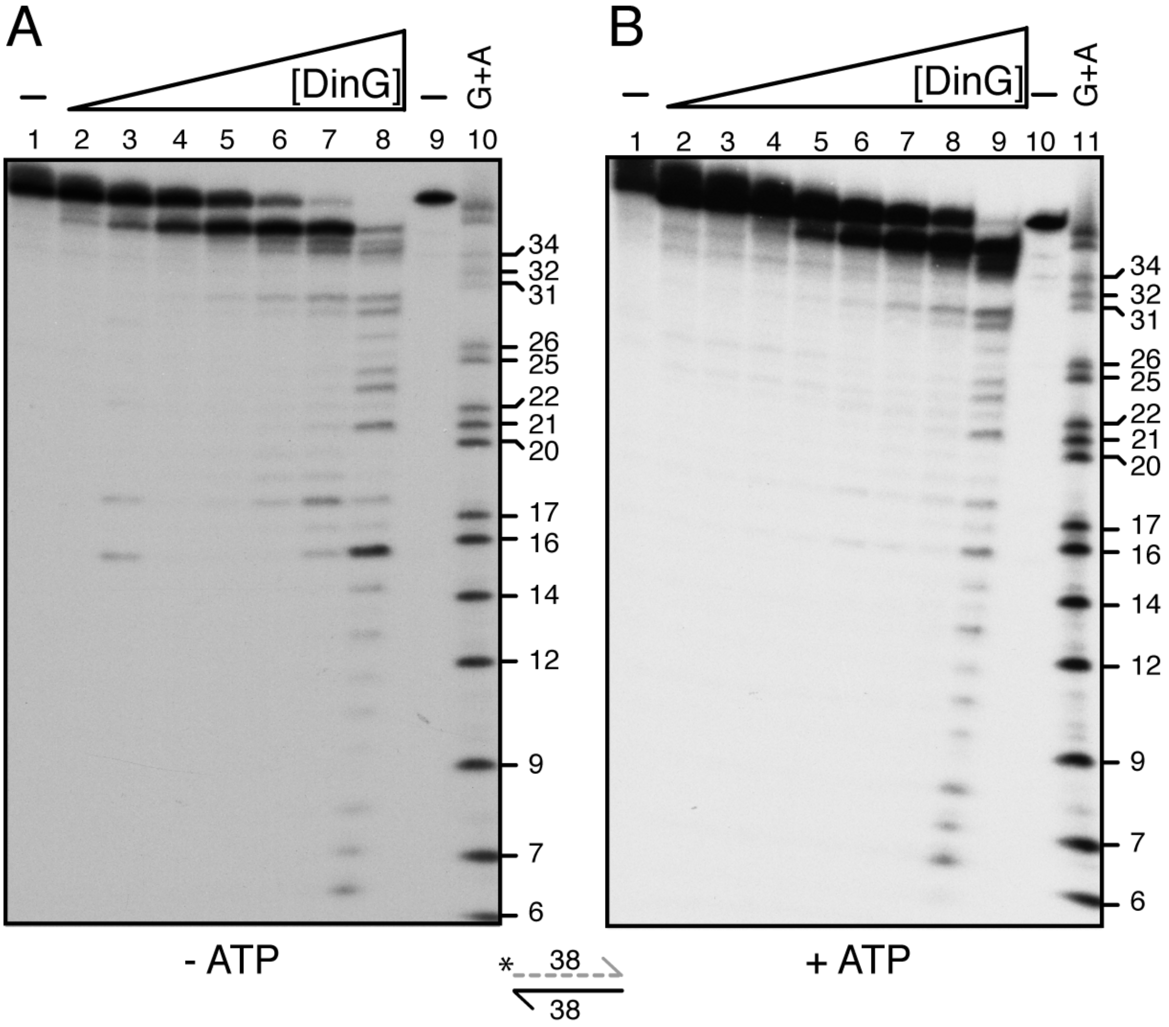

Figure S6. DinG degrades blunted RNA─DNA hybrids. (**A-B**) Duplex γ^{32}P-RNA-DNA (5 nM in DNA molecules) was incubated with increasing concentrations of DinG (0.3─25 and 100 nM [**A**] or 0.15─25 and 100 nM [**B**]) for 15 min at 37 ºC in buffer D, either in the absence (**A**) or presence of 2.5 mM ATP (**B**). Reaction products were then separated under denaturing conditions. "–", no protein added; grey line, 5´-end labelled strand; "*", labelled nucleotide; arrowhead, 3´-end; solid line, DNA; dashed line, RNA; number, length of each strand (in nt); and "G + A", mobility marker, with the molecule lengths indicated in nt (lanes 10 and 11). All reactions were repeated at least three times with consistent results, and representative gel images are shown.

A

Relevant genotype	Chromosomal transformation	Plasmid transformation
rec^{+}	1 (1.5 x 10^{-5})	1 (2.4 x 10^{-6})
+ Δ*dinG*	0.9 ± 0.2	0.4 ± 0.1
+ Δ*recJ*	0.09 ± 0.04	0.8 ± 0.3
+ Δ*dinG* Δ*recJ*	0.1 ± 0.1	0.4 ± 0.2
+ Δ*recQ*	0.27 ± 0.1	0.5 ± 0.2
+ Δ*dinG* Δ*recQ*	0.21 ± 0.1	0.5 ± 0.2
+ Δ*recS*	0.9 ± 0.3	0.8 ± 0.1
+ Δ*dinG* Δ*recS*	0.9 ± 0.4	0.3 ± 0.1
+ Δ*addAB*	0.8 ± 0.1	0.9 ± 0.1
+ Δ*dinG* Δ*addAB*	0.8 ± 0.2	0.8 ± 0.1

B

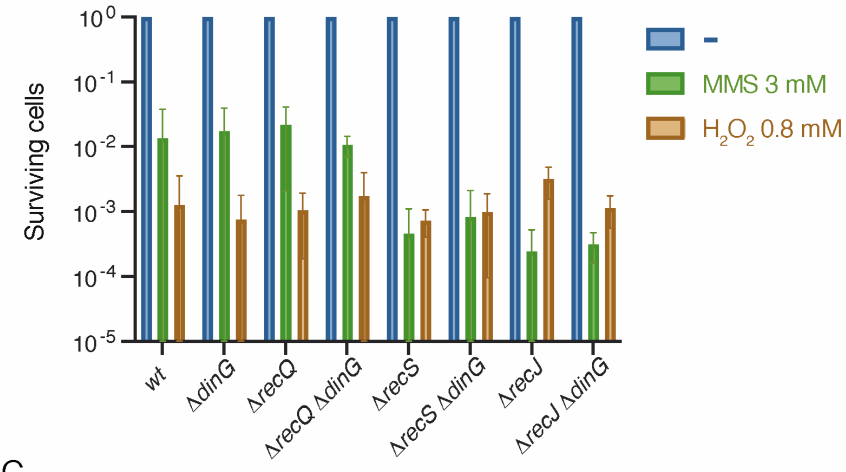

C

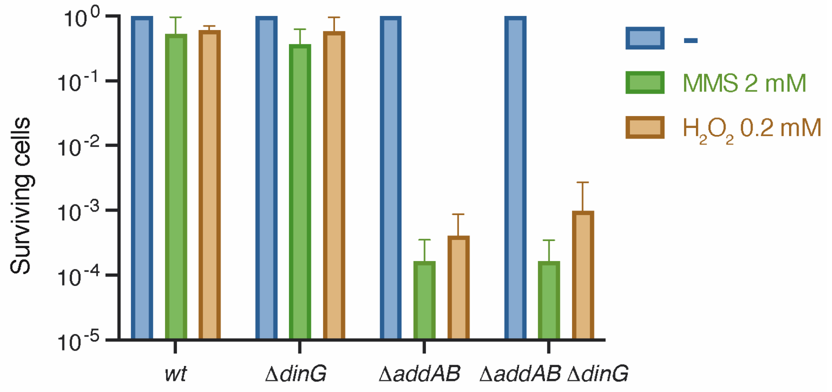

Figure S7. DinG does not contribute to end-resection. (**A**) The indicated strains were made competent and transformed with 0.1 µg·ml^{-1} of homologous *rpoB*482 DNA (Rif^{R}, 8 µg·ml^{-1}) or heterologous pHP14 plasmid DNA (Ery^{R}, 2 µg·ml^{-1}) as described (see Material and methods). Transformation efficiency was assessed by normalizing the yields of Rif^{R} (chromosomal transformants) and Nm^{R} (plasmid transformants) CFUs relative to that of the wt strain, recorded as 1 (in parentheses, number of transformants / total number of cells), as described [16]. (**B**–**C**) The indicated strains were grown to exponential phase (OD_{560} = 0.4) in LB at 37 ºC, serially diluted, and plated on LB plates containing the specified concentration of MMS or H_2O_2. Plates were incubated ON at 37 ºC, and results are plotted as CFUs upon exposure to MMS or H_2O_2 normalised to those obtained from untreated cells, recorded as 1. Results are the mean ± SEM of >3 independent experiments.

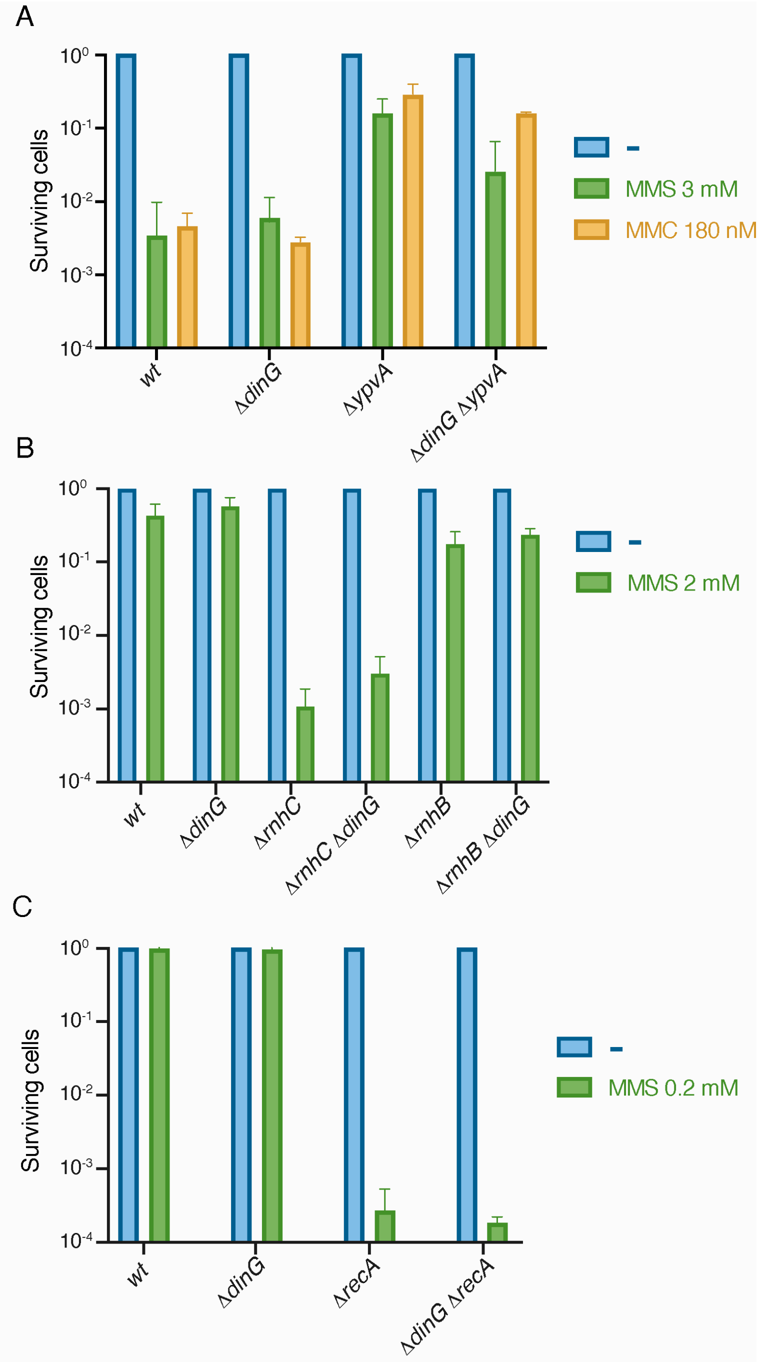

Figure S8. Survival of the Δ*dinG* mutant in the absence of RnhB, RnhC, or RecA. The indicated strains were grown to exponential phase (OD_{560} = 0.4) in LB medium at 37 ºC, serially diluted, and plated on LB plates containing the specified concentration of MMS. Plates were incubated ON at 37 ºC, and results are plotted as CFUs upon exposure to MMS normalised to those obtained from untreated cells, recorded as 1. Results are the mean ± SEM of >3 independent experiments.

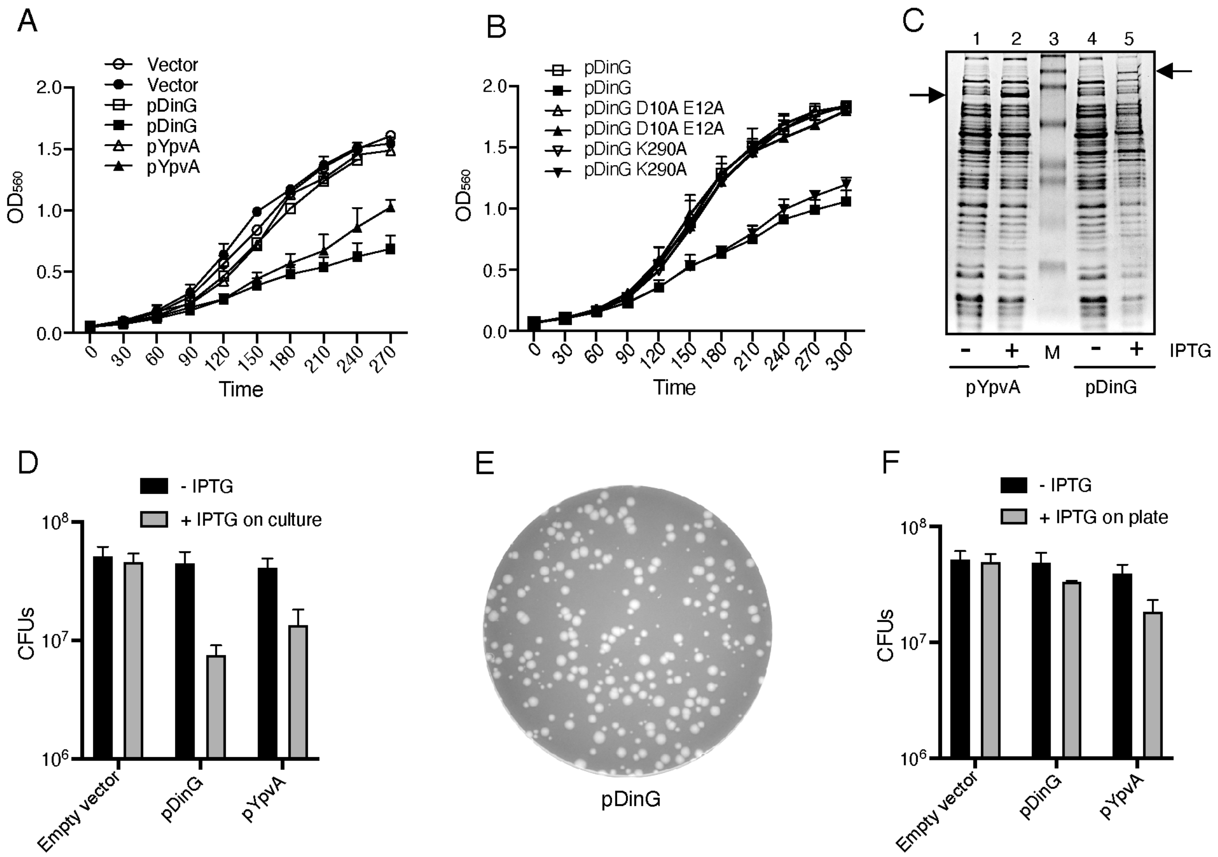

Figure S9. Effect of DinG and YpvA overexpression on cell proliferation. (**A-B**) Cells harbouring the empty vector, pDinG, pYpvA, pDinG D10A E12A, or pDinG K290A were grown in LB medium with Nm, and either without (empty symbols) or with 125 μM IPTG (filled symbols). The increase in OD_{560} was measured across three independent experiments, and mean values were plotted. (**C**) Crude extracts from cells harbouring pDinG or pYpvA were analysed by SDS-PAGE following growth in LB medium without (-) or with 125 μM IPTG (+). The position of YpvA and DinG proteins is indicated by black arrows. (**D**) Cells bearing the empty vector, pDinG, or pYpvA were grown to an OD_{560} = 0.4 in LB medium with or without 125 μM IPTG. Then IPTG was then removed, cells were serially diluted, and plated on LB agar. Viable cell counts were performed in three independent experiments, and mean values are plotted. (**E**) A representative plate showing normal-sized and small-sized colonies of cells bearing pDinG is shown. (**F**) Cells containing the empty vector, pDinG, or pYpvA were grown in LB medium to an OD_{560} = 0.4 and plated on LB agar with or without 125 μM IPTG. Viable cells count was performed in three independent experiments, and mean values are plotted.

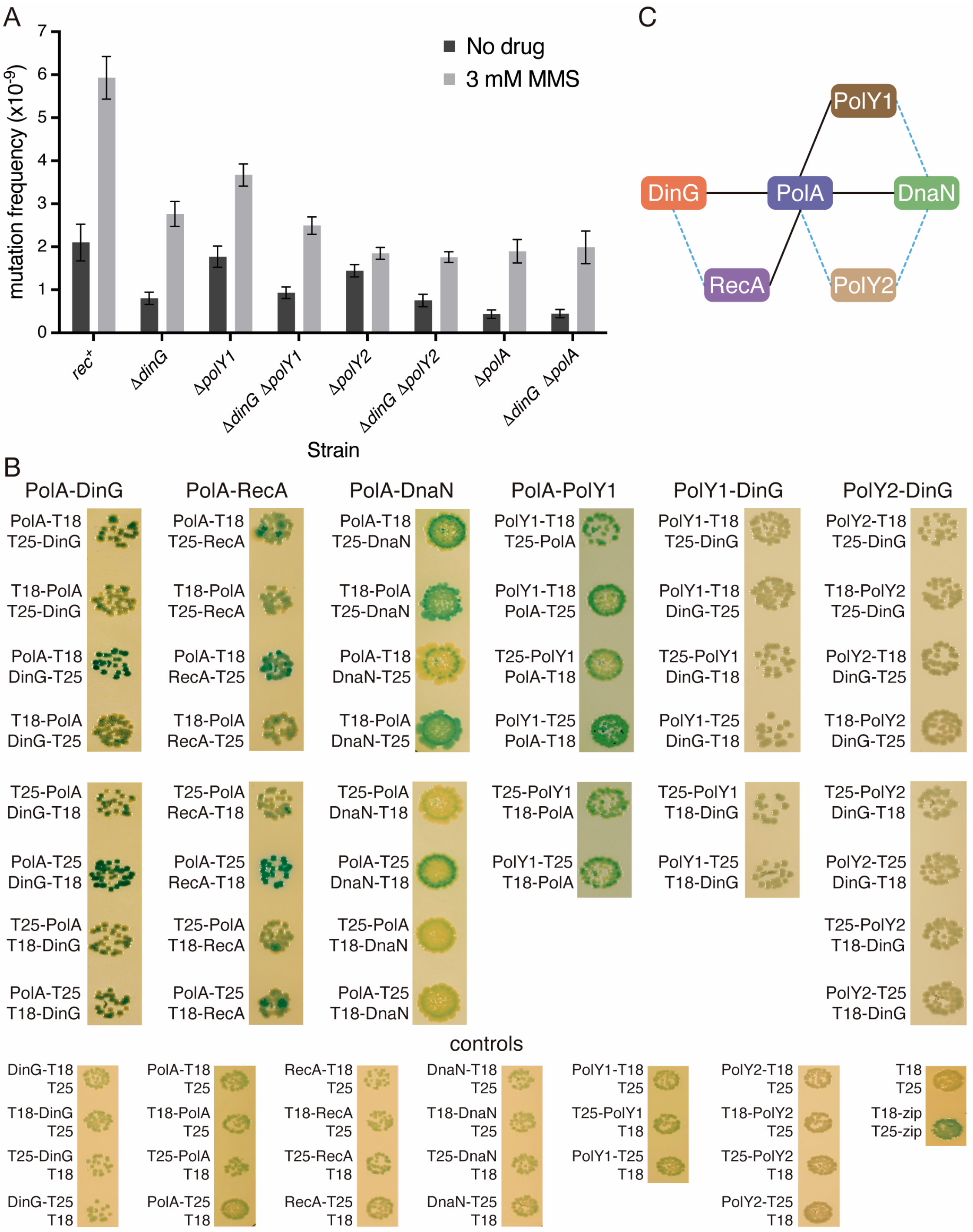

Figure S10. Role of DinG in mutagenesis. Absence of DinG reduces spontaneous and damage-induced mutations. (A) *rec*+, Δ*dinG*, Δ*polY1*, Δ*polY2*, Δ*dinG* ΔpolY1, Δ*dinG* Δ*polY2*, Δ*polA*, and Δ*dinG* Δ*polA* cells were grown to an OD_{560} = 0.8 in LB medium. A 10 ml aliquot of each culture was exposed to 3 mM MMS (grey bar) for 15 min. Cultures were then plated on LB or LB containing rifampicin, and mutation frequency was scored. Data are represented as the mean ± SD from at least four independent experiments. (B and C) PolA interacts with DinG, PolY1, DnaN, and RecA. (B) Interactions between full-length proteins fused to the N- or C-terminal of the T18 or T25 domains were assayed by the bacterial two-hybrid system. (C) Diagram illustrating the physical interactions identified in this study between PolY1, PolY2, Pol I, and DnaN (solid lines), and additional physical or functional interactions revealed by the yeast two-hybrid system or physical interaction among purified proteins (dotted lines).

References

1. Lovett, S. T., The DNA Exonucleases of *Escherichia coli*. *EcoSal Plus* 2011, 4, (2).
2. Sanchez, H.; Kidane, D.; Cozar, M. C.; Graumann, P. L.; Alonso, J. C., Recruitment of *Bacillus subtilis* RecN to DNA double-strand breaks in the absence of DNA end processing. *J Bacteriol* 2006, 188, (2), 353-60.
3. Dubnau, D.; Blokesch, M., Mechanisms of DNA Uptake by Naturally Competent Bacteria. *Annu Rev Genet* 2019.
4. Maier, B., Competence and Transformation in *Bacillus subtilis*. *Curr Issues Mol Biol* 2020, 37, 57-76.
5. Carrasco, B.; Serrano, E.; Sanchez, H.; Wyman, C.; Alonso, J. C., Chromosomal transformation in *Bacillus subtilis* is a non-polar recombination reaction. *Nucleic Acids Res* 2016, 44, (6), 2754-68.
6. Serrano, E.; Ramos, C.; Alonso, J. C.; Ayora, S., Recombination proteins differently control the acquisition of homeologous DNA during *Bacillus subtilis* natural chromosomal transformation. *Environ Microbiol* 2021, 23, (1), 512-524.
7. Sedgwick, B., Repairing DNA-methylation damage. *Nat Rev Mol Cell Biol* 2004, 5, (2), 148-57.
8. Friedberg, E. C.; Walker, G. C.; Siede, W.; Wood, R. D.; Schultz, R. A.; Ellenberger, T., *DNA repair and mutagenesis*. ASM Press: Washington DC, 2005.
9. Moreno-del Alamo, M.; Carrasco, B.; Torres, R.; Alonso, J. C., *Bacillus subtilis* PcrA Helicase Removes Trafficking Barriers. *Cells* 2021, 10, (4).
10. Carrasco, B.; Moreno-del Álamo, M.; Torres, R.; Alonso, J. C., PcrA Dissociates RecA Filaments and the SsbA and RecO Mediators Counterbalance Such Activity. *Front Mol Biosci* 2022, 9, 836211.
11. Torres, R.; Romero, H.; Rodriguez-Cerrato, V.; Alonso, J. C., Interplay between *Bacillus subtilis* RecD2 and the RecG or RuvAB helicase in recombinational repair. *DNA Repair (Amst)* 2017, 55, 40-46.
12. Fujita, M.; Sadaie, Y., Rapid isolation of RNA polymerase from sporulating cells of *Bacillus subtilis*. *Gene* 1998, 221, (2), 185-90.
13. Stragier, P.; Bonamy, C.; Karmazyn-Campelli, C., Processing of a sporulation sigma factor in *Bacillus subtilis*: how morphological structure could control gene expression. *Cell* 1988, 52, (5), 697-704.
14. Carrasco, B.; Ayora, S.; Lurz, R.; Alonso, J. C., *Bacillus subtilis* RecU Holliday-junction resolvase modulates RecA activities. *Nucleic Acids Res* 2005, 33, (12), 3942-52.
15. Karimova, G.; Pidoux, J.; Ullmann, A.; Ladant, D., A bacterial two-hybrid system based on a reconstituted signal transduction pathway. *Proc Natl Acad Sci U S A* 1998, 95, (10), 5752-6.
16. Alonso, J. C.; Luder, G.; Tailor, R. H., Characterization of *Bacillus subtilis* recombinational pathways. *J Bacteriol* 1991, 173, (13), 3977-80.
